# Novel combination therapy for melanoma induces apoptosis via a gap junction positive feedback mechanism

**DOI:** 10.18632/oncotarget.27732

**Published:** 2020-09-15

**Authors:** Archis Bagati, Timothy C. Hutcherson, Zethan Koch, Joseph Pechette, Hossein Dianat, Cory Higley, Lisa Chiu, Yesul Song, Jay Shah, Elana Chazen, Andrew Nicolais, Peter Casey, Kyle Thompson, Kevin Burke, Mikhail A. Nikiforov, Jennifer Zirnheld, Shoshanna N. Zucker

**Affiliations:** ^1^Department of Cell Stress Biology, Roswell Park Comprehensive Cancer Center, Buffalo, New York, USA; ^2^Department of Pharmacy Practice, D’Youville School of Pharmacy, Buffalo, New York, USA; ^3^Department of Pharmaceutical, Social, and Administrative Sciences, D’Youville School of Pharmacy, Buffalo, New York, USA; ^4^Department of Electrical Engineering, University at Buffalo, Buffalo, New York, USA; ^*^These authors contributed equally to this work and share first authorship

**Keywords:** non-thermal plasma, gap junction, tirapazamine, melanoma, intercellular communication

## Abstract

Metastatic melanoma cells overexpressing gap junctions were assayed for their ability to propagate cell death by a novel combination therapy that generates reactive oxygen species (ROS) by both 1) non-thermal plasma (NTP) and 2) tirapazamine (TPZ) under hypoxic conditions. Results demonstrate additive-to-synergistic effects of combination therapy compared to each agent individually. NTP induces highly localized cell death in target areas whereas TPZ partially reduces viability over the total surface area. However, when high gap junction expression was induced in melanoma cells, effects of combination NTP+TPZ therapy was augmented, spreading cell death across the entire plate. Similarly, *in vivo* studies of human metastatic melanoma in a mouse tumor model demonstrate that the combined effect of NTP+TPZ causes a 90% reduction in tumor volume, specifically in the model expressing gap junctions. Treatment with NTP+TPZ increases gene expression in the apoptotic pathway and oxidative stress while decreasing genes related to cell migration. Immune response was also elicited through differential regulation of cytokines and chemokines, suggesting potential for this therapy to induce a cytotoxic immune response with fewer side effects than current therapies. Interestingly, the gap junction protein, Cx26 was upregulated following treatment with NTP+TPZ and these gap junctions were shown to maintain functionality during the onset of treatment. Therefore, we propose that gap junctions both increase the efficacy of NTP+TPZ and perpetuate a positive feedback mechanism of gap junction expression and tumoricidal activity. Our unique approach to ROS induction in tumor cells with NTP+TPZ shows potential as a novel cancer treatment.

## INTRODUCTION

Melanoma is a growing problem in many parts of the world due to a reduced ozone layer and increased exposure to natural and artificial solar radiation [1]. Although melanoma only accounts for 1.5% of all cancer-related mortality, it causes 5.3% of all newly diagnosed cases of cancer and is responsible for most skin-related cancer deaths. In 2020, it is estimated that there will be 100,350 new cases and 6,850 deaths in the United States from melanoma [1].

Current treatment options focus on two primary approaches; namely, gene targeting and immunotherapy. Gene targeting therapies, with medications like vemurafenib, target a BRAF600 mutation which is found in approximately 50% of melanoma patients [2]. This approach is often coupled with the MEK inhibitor, cobimetinib [3]. Even with combination therapy there is a high degree of resistance and recurrence, which is often fatal [4].

Immunotherapy is showing significant progress in melanoma. Recent success has been demonstrated with ipilimumab (a checkpoint inhibitor to the CTLA-4 pathway), as well as pembrolizumab and nivolumab, which are PD-1 checkpoint inhibitors [5]. Despite the success of these and related compounds, there are potential side effects including damage to normal organs and tissues, sometimes leading to death [6]. There is a need for improved therapy which is both highly effective and reduces side effects. To this end, we have demonstrated that a novel, patented combination therapy is highly selective for treating cancer cells and shows promising effects both *in vitro* and *in vivo* [7] with the ability to differentially regulate cytokines and chemokines as demonstrated in this study. However, due to the specificity for cancer cells, this treatment may offer a therapeutic advantage. The novel combination therapy includes an electrical component, non-thermal plasma (NTP), and a DNA-damaging pharmaceutical agent, tirapazamine (TPZ).

NTP, also known as cold plasma or atmospheric plasma, generates plasma at room temperature. The plasma utilized in these studies is created with the addition of helium gas that is ionized and mixed with atmospheric gases as it flows across a high electric potential [8]. It has proven effective in various biomedical applications, such as wound healing, coagulation, sterilization, and in the treatment of cancer [9]. NTP has been shown to cause apoptosis in melanoma cells by both the dielectric barrier discharge (DBD) method using two electrodes and the method of a plasma torch whereby the tissue surface serves as the second electrode [8, 10, 11]. The NTP torch utilized in these studies has been shown to generate 45 species, including both inert and charged molecules [12]. NTP is a source of both reactive oxygen species (ROS) and reactive nitrogen species (RNS) [13]. ROS have been shown to increase activation of mitogenic signaling pathways, while also promoting further increase in ROS production and induction of apoptosis [14, 15].

Thus far we have demonstrated that NTP causes selective apoptotic cell death in melanoma cells [16]. We investigated mechanisms to enhance the cytotoxicity of NTP by using innate properties of cancer cells, such as hypoxia. Due to the hypoxic environment of tumors, we tested the effect of a prodrug, TPZ, that undergoes a structural change to an active entity under hypoxia [17, 18]. In hypoxic conditions, it enzymatically exchanges a hydroxyl group for an oxygen radical, becoming active as a ROS molecule [19]. TPZ breaks double-stranded DNA and is also a topoisomerase II inhibitor that prevents DNA repair [18]. TPZ has been used in phase 3 clinical trials for non-small-cell lung, cervical, and head and neck cancers. Furthermore, it has also been evaluated in phase 2 clinical trials for the aforementioned disease states and glioblastoma, ovarian, primary peritoneal cancer, and melanoma [20]. Its efficacy appears limited when used as a single agent or in combination with cisplatin, carboplatin, paclitaxel, or irradiation; but TPZ has been shown to be generally tolerable in humans [21]. TPZ is not currently approved by the US Food and Drug Administration for therapeutic use [22]. However, in our application, we demonstrate that the NTP+TPZ combination therapy increases both apoptosis and the oxidative stress response without the need for additional chemotherapeutic agents [16]. To target the maximum tumor volume, we sought to increase the target area of treatment.

We have previously demonstrated that gap junctions expand the area of cell death affected by NTP [16]. Gap junctions promote intercellular communication through the transfer of charged and neutral species of up to 1 kilodalton (kDa) including ROS, RNS, and other free radicals [23]. Gap junctions enable the transport of ROS molecules between cells via the bystander effect as demonstrated by radiation treatment [24]. Transfer of ROS promotes cellular cytotoxicity to unirradiated bystander cells in a gap junction dependent manner. In addition, chemotherapeutic agents, such as cisplatin, have been demonstrated to enhance the bystander effect by the drug itself going through the gap junctions [25].

Multiple molecules generated by non-thermal plasma and their derivative molecules as well as the TPZ itself qualify as potential permeants that can be passed between cells via the bystander mechanism. Thus, we tested the effects of this combination therapy. In order to determine the role of cell communication on therapeutic efficacy, we utilized metastatic melanoma (1205Lu) cells that we previously generated to overexpress either 1) wild type connexin 43 (1205LuC), 2) a dominant negative connexin 43 that blocks both coupling with the mutant as well as wild type coupling through heterotypic gap junction channels (1205LuT) as well as the plasmid control (1205LuP) which has very low endogenous coupling [26]. These cell lines all express an IRES vector linking GFP to connexin expression and were selected through three rounds of cell sorting to be 100% GFP expressing. The cell lines differ only in their expression of functional gap junctions and, thus, we were able to assess the specificity of gap junction functionality in this study. The cells have previously been characterized for their properties and response to NTP [27, 28]. In this study we address the combination therapy of non-thermal plasma and TPZ on metastatic melanoma and the role of gap junctions in potentiating the therapeutic response.

## RESULTS

### Independent effects of NTP and TPZ on cell viability

**Figure 1:**
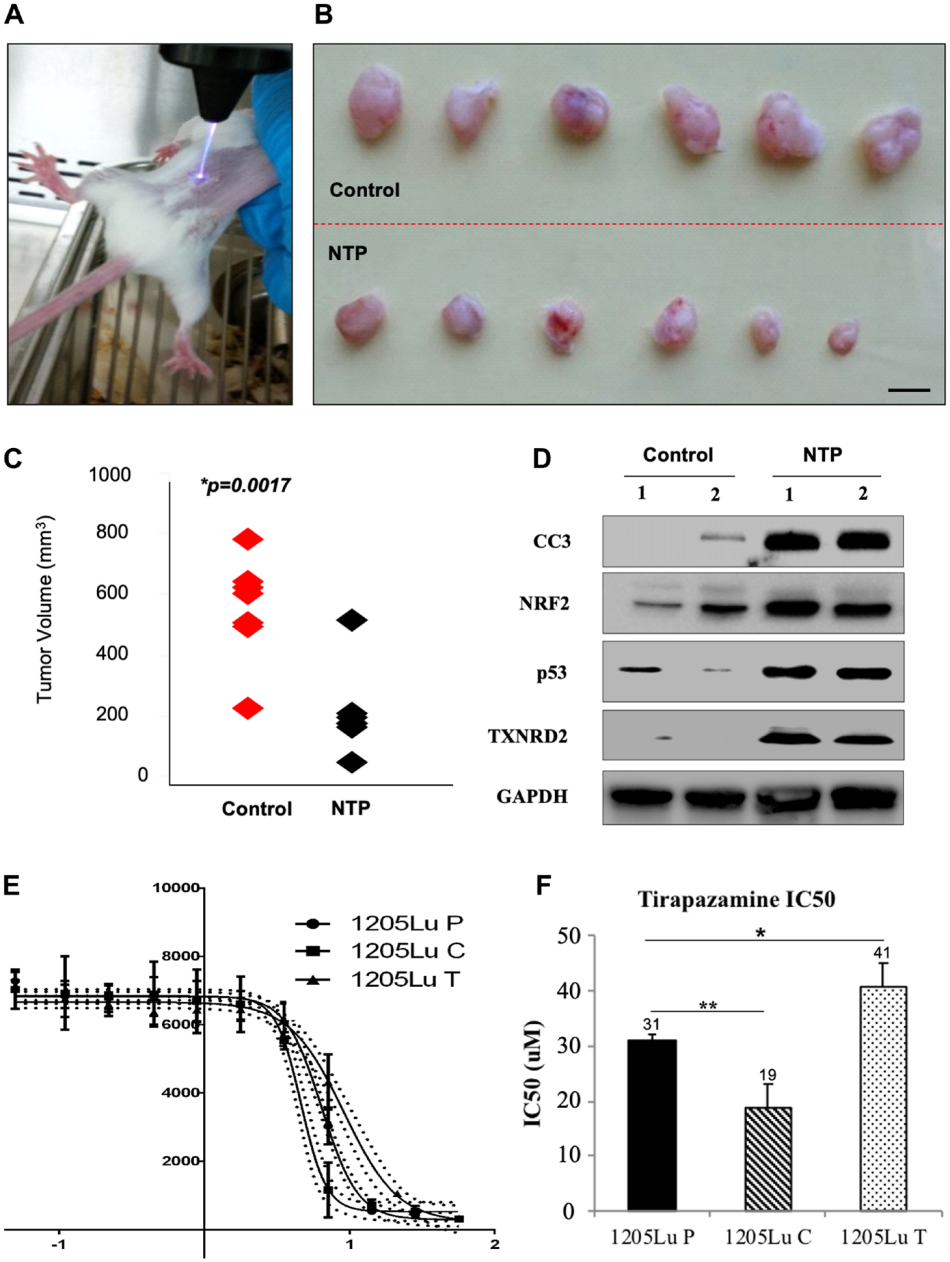
Analysis of effects of NTP and TPZ independently. (**A**) *In vivo* treatment of plasma torch on 1205Lu C human metastatic melanoma tumor in a SCID mouse. (**B**) Top panel: untreated tumors excised from SCID mice, Bottom panel: treated with NTP for 30 sec. (**C**) Comparison of the range of tumor sizes, control in red and NTP treated in black, *p* = 0.017. (**D**) Assessment of gene expression changes in the control and NTP-treated tumor samples by RT-PCR. These include cleaved caspase 3 (CC3), Nuclear factor (erythroid-derived 2)-like 2 (NRF2), p53, Thioredoxin reductase 2 (TXNRD2). (**E**) Log dose response curve for TPZ in the cell lines, 1205LuP (1205Lu cells with IRES GFP plasmid), 1205LuC (1205Lu cells with IRES GFP plasmid expressing Cx43), and 1205LuT (1205Lu cells with IRES GFP plasmid expressing a dominant negative Cx43 mutation, T154A) to determine the IC_50_ dose. (**F**) IC_50_ dose for 1205LuP = 31 μM, 1205LuC = 19 μM, 1205LuT = 41 μM. Statistical variances using the unpaired Student’s *t* test are compared as indicated (^*^ *p* < 0.05; ^**^ *p* < 0.01).

Due to the previous *in vitro* demonstration that NTP induces apoptosis in melanoma cells, we investigated the role of NTP on tumor volume in a mouse model where the tumors were treated directly with the NTP torch (Figure 1A) [10, 16]. The tumors were measured and grouped to compare the control and NTP-treated tumors (Figure 1B). There was a highly significant decrease in tumor volume in the NTP-treated group as compared to the untreated control group (*p* = 0.0017) (Figure 1C).

To compare the protein levels between untreated and NTP-treated tumor samples, lysates were prepared from homogenized tumors for Western blot analysis. As compared to the untreated samples, the NTP-treated samples showed an increase in several proteins including the apoptotic protein, cleaved caspase 3 (CC3) (Figure 1D), consistent with previous reports of NTP-induced apoptosis [16]. Furthermore, there was an increase in NRF2, p53, and TXNRD2 (Figure 1D), which are all indicative of an increase in oxidative stress.

**Figure 2:**
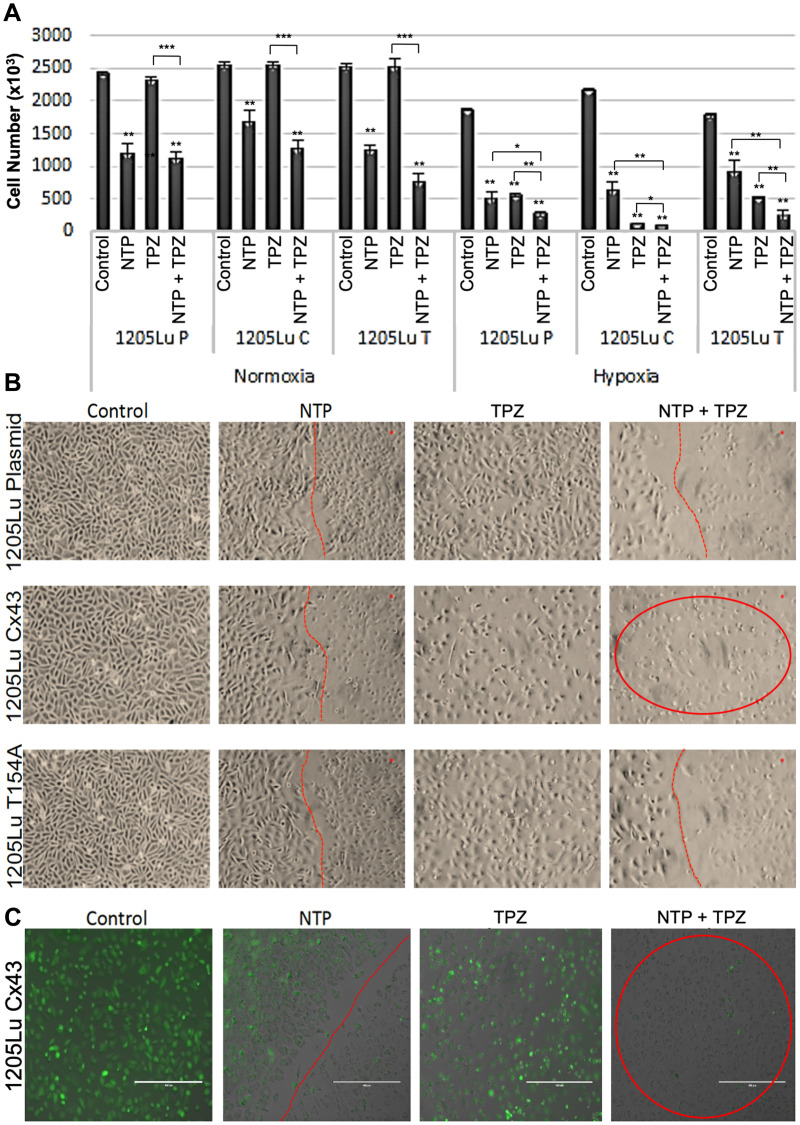
Effects of combination gap junctions on NTP+TPZ efficacy. (**A**) Results of viability assay under normoxia or hypoxia conditions with NTP, 20 μM TPZ, NTP+20 μM TPZ with the cell lines 1205LuP, 1205LuC, 1205LuT. Statistical variances using the unpaired Student’s *t* test are compared to controls unless otherwise indicated (^*^ *p* < 0.05, ^**^ *p* < 0.01, and ^***^ *p* < 0.001). (**B**) Phase microscopic images of cells treated with NTP (cells right of the red line are NTP-treated), TPZ, NTP + TPZ (cells in circle for 1205LuC shows loss of viability). (**C**) By fluorescent microscopy, we document the live cells that express GFP (green) and the dead cells that lose GFP expression. Note that images were taken at the live/dead border to document the area treated by NTP (to the right of the dotted red line). Figure 2C highlights the 1205LuC cells that lose all GFP expression with NTP+TPZ (red circle). (scale bar = 400 μm).

Since the tumor environment is typically hypoxic, we compared the effects of NTP-treated melanoma cells at normoxia and hypoxia [29]. We demonstrated a decrease in cell proliferation under hypoxic conditions for both the untreated and NTP-treated melanoma cells (Figure 2A). Due to this effect, we chose to combine NTP treatment with a DNA damaging agent that was activated under hypoxic conditions and was already in clinical trials, TPZ. It has previously been demonstrated that when maintained in a hypoxic chamber, 1205Lu cells express significantly altered levels of hypoxia markers including increased expression of *MXI1*(max interactor 1) and decreased expression of both *MCAM* (melanoma cell adhesion molecule) and *NME1* (non-metastatic cells 1) which are characteristic of hypoxic cells [29].

We tested the IC_50_ for TPZ in 1205Lu melanoma cells expressing an empty plasmid and has low gap junction coupling (GJIC), (1205LuP), overexpressing wild type connexin43 which have high GJIC, (1205LuC), and overexpressing a dominant negative connexin 43 which have essentially no GJIC, (1205LuT) (Figure 1E, 1F). All cells have been previously characterized [27]. Of the 11 connexins expressed in skin, Cx26, Cx30, Cx30.3, Cx31, Cx31.1, Cx32, Cx36, Cx37, Cx40, Cx43, and Cx45, there was no detectable expression for Cx26, Cx30, Cx31.1, Cx36, and Cx37 in parental 1205Lu cells, low expression for connexins 30.3, and 31 and higher expression levels for connexins 32, 40, 43, and 45, of which only Cx43 was upregulated when the cells were migrating. For this reason, we overexpressed a wild type or dominant negative Cx43 in 1205Lu cells whereby the protein levels of Cx43 for the wild type Cx43 (1205LuC) and T154A (1205LuT) increased by 25-30-fold [27].

We also utilized the fact that the structure and size of TPZ suggests that it could pass through gap junctions. By dose response curves, we calculated the IC_50_ for TPZ: 1205LuP = 31 micromolar (μM), 1205LuC = 19 μM, and 1205LuT = 41 μM. Thus, the lowest IC_50_ was demonstrated for the most highly coupled cells (Figure 1F). This suggests that gap junction intercellular communication (GJIC) may play an important role in the cell killing effect. A premetastatic melanoma cell line from which 1205Lu was derived, WM793B, was also tested with an IC_50_ of 26 μM. In addition, cervical carcinoma and colon cancer cell lines showed a similar IC_50_ range to the 1205Lu series (Supplementary Figure 1).

### Combined effects of NTP+TPZ on cell viability

To test the combined effects of NTP and TPZ as well as a potential effect of gap junctions, we treated melanoma cells with NTP alone, TPZ alone, or the combined treatment with NTP+TPZ [7]. Figure 2A shows the cell viability under conditions of normoxia and hypoxia. Note that in normoxia, the TPZ has no effect, whereas it has a cytotoxic effect at hypoxia. This is due to the established structural change of TPZ to its active form only under hypoxic conditions [19]. Cell viability in the NTP+TPZ combination treatment was significantly decreased as compared to each component alone. The only exception is for the 1205LuC cells in which the cells are almost completely dead for the TPZ as well as the NTP+TPZ (Figure 2B, red circle). This is due to the lower IC_50_ for the TPZ in this cell line as compared to the others (Figure 1F). This experiment is representative of five similar experiments, some of which were examined with a fluorescent microscope (Figure 2C, Supplementary Figure 2). To compare the live cells that express GFP (green) to the dead cells lose GFP expression, we recorded images along the live/dead border to document the area treated by NTP. Figure 2C highlights that the 1205LuC cells were the only cell line that lost all GFP expression with NTP+TPZ (red circle).

In parallel, we assessed cell viability both quantitatively (Figure 2A) and qualitatively (Figure 2B). For all three cell lines, the cells under control conditions showed high viability. The NTP-treated cells to the left of the red line showed high viability, whereas those directly treated with NTP (cells to the right of the red line) showed increased dead cells. This confirmed our studies with GFP as a marker for cell viability [28]. TPZ caused an extent of cell death throughout the plate that correlated with the drug dose versus IC_50_ for each cell line. However, the most compelling result was that when all three cell lines were treated with the combination therapy, only the 1205LuC cells demonstrated all dead cells throughout the 10-centimeter (cm) dish (Figure 2B, circled in red). The results showed a complete loss of the line demarcating the area of plasma treatment with untreated cells (as shown by the red line in other images). Thus, the entire plate showed dead cells. This result was highly reproducible (five repetitions) [7]. The spreading of the cell death signals for NTP+TPZ was only observed for 1205LuC cells in all repetitions of the same experiment (Figure 2B, 2C, Supplementary Figure 2), suggesting that gap junctions may play a role in the promotion of cell death signals by the bystander method [24].

**Figure 3:**
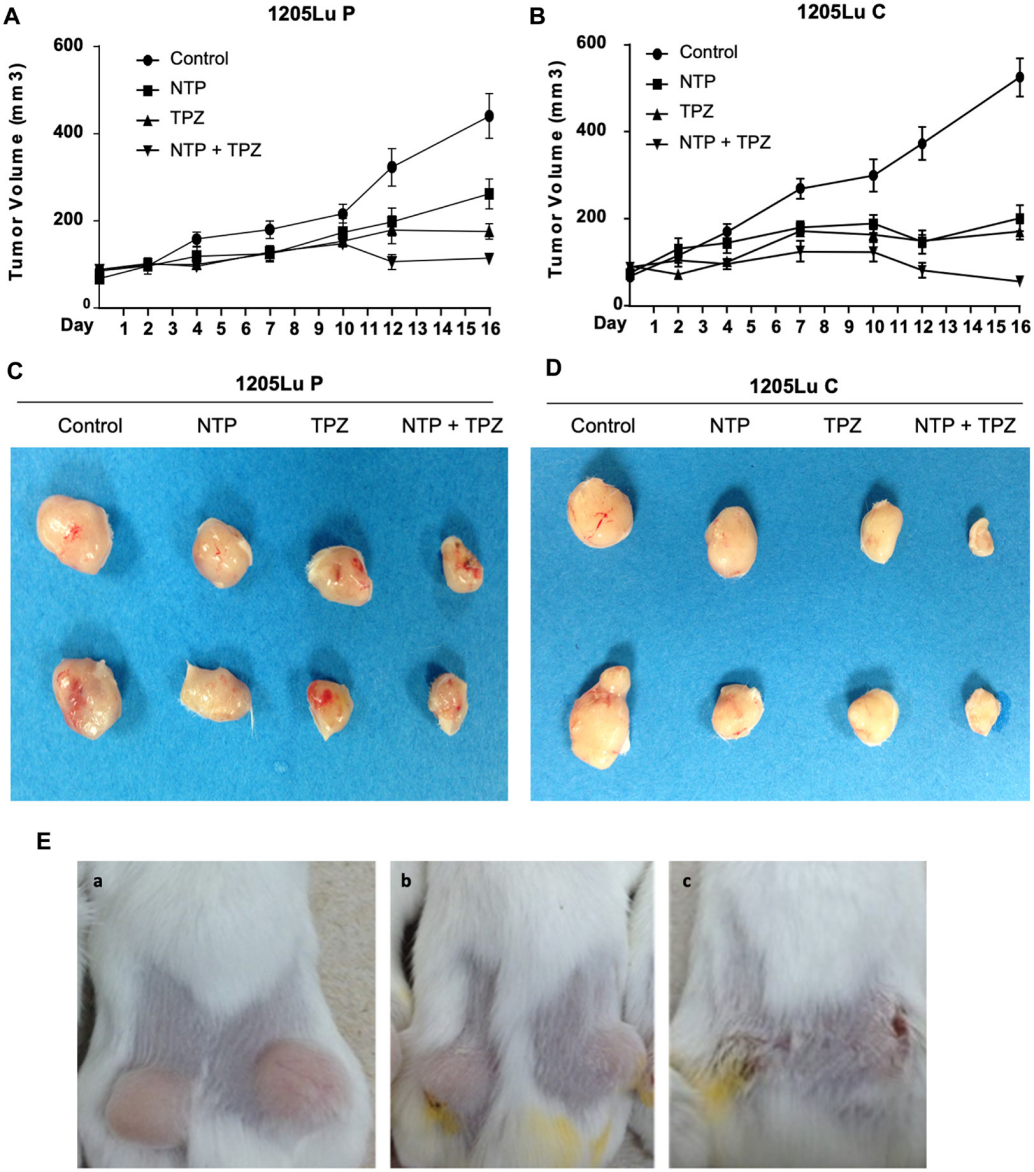
Effects of gap junctions on *in vivo* treatment with NTP+TPZ. (**A**) Tumors grown in mice from1205LuP cells were treated every two or three days for five treatments (completed by day 10 and recorded until day16). Significance is detailed in the Results section. The 1205LuC tumors show a significant change in tumor volume at an earlier time point (by day 4) for TPZ (*p* = 0.0059) and NTP+TPZ (0.0066) and NTP (*p* = 0.0391) whereas the 1205LuP tumors did not show significance until day 10 for TPZ (*p* = 0.0296) and NTP+TPZ (*p* = 0.0122) and day 12 for significance of NTP (*p* = 0.0344). (**B**) Tumors grown in mice from1205LuC cells were treated every two or three days for five treatments (completed by day 10 and recorded until day 16). Statistical analyses for a and b are in the text. (**C**) Representative 1205LuP tumor samples at day 16 from control, NTP, TPZ, NTP+TPZ. (**D**) Representative 1205LuC tumor samples at day 16 from control, NTP, TPZ, NTP+TPZ. (**E**) Mice with tumors from 1205LuC cells under conditions of Control (Panel A), TPZ treated (Panel B), NTP+TPZ treated (Panel C).

The results of *in vivo* SCID xenograft mouse models demonstrate that tumor growth was inhibited in the xenografts from both 1205LuP and 1205LuC cell lines; however, there was a more significant decrease in the 1205LuC cells (Figure 3A, 3B). When comparing the *p* values during each time point between 1205LuP and 1205LuC, it is notable that the 1205LuC tumors show a significant change in tumor volume at an earlier time point (highly significant by day 4 for TPZ (*p* = 0.0059) and NTP+TPZ (0.0066) and significant for NTP (*p* = 0.0391). By day 7 all conditions were highly significant including NTP (*p* = 0.0082), TPZ (*p* = 0.0001), and NTP+TPZ (*p* < 0.0001). By day 16 all conditions were all extremely significant for 1205LuC NTP (38.3% of total volume), TPZ (32.5% of total volume), and NTP+TPZ (10.8% of total volume), all at (*p* < 0.0001).

The 1205LuP tumors did not show significance until day 10 for TPZ (*p* = 0.0296) and NTP+TPZ (*p* = 0.0122) and day 12 for significance of NTP (*p* = 0.0344) with high significance of NTP+TPZ (*p* = 0.0004). By day 16, the *p* values for 1205LuP were the following: NTP (59.5% of total volume) (*p* = 0.0115), TPZ (39.9% of total volume) (*p* = 0.0011), NTP = TPZ (25.8% of total volume) (*p* < 0.0001). This data was representative of three experiments. The decrease in tumor volume with NTP+TPZ was observed for the 1205LuP and 1205LuC yet was most apparent for 1205LuC where a sample of the tumors are shown (Figure 3C, 3D). This observation was confirmed in several experiments, as well as in examination of the tumors in the mice as shown for 1205LuC (Figure 3E). The saline injections or uninjectable control tumors were not statistically variable (Supplementary Figure 3).

### Gene expression changes associated with NTP+TPZ treatment

**Figure 4:**
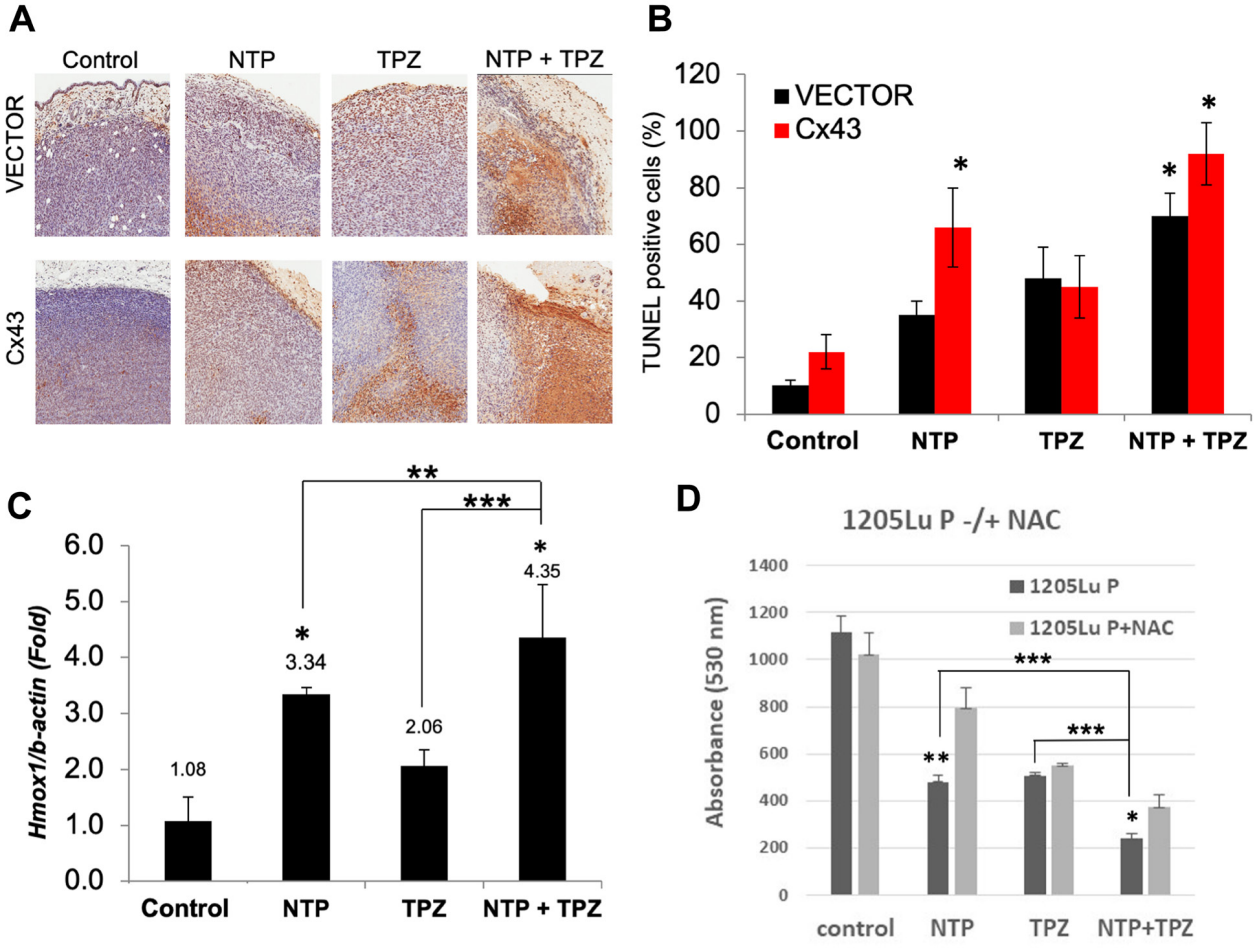
Analysis of apoptosis and oxidative stress on *in vivo* treatment with NTP+TPZ. (**A**) TUNEL assay is indicative of apoptosis as shown by the brown staining in histological sections. (**B**) Quantification of TUNEL staining from multiple histological sections for 1205LuP (vector) tumors and 1205LuC (Cx43) tumors (^*^ *p* < 0.05). (**C**) Analysis of RT-PCR from homogenized 1205LuC tumors that were control or treated with NTP, TPZ, or NTP+TPZ. 100 cells were counted per condition. with coupling ranging from 0% (untreated) to 5% Statistical variances using the unpaired Student’s *t* test are compared to controls unless otherwise indicated (^*^ *p* < 0.05, ^**^ *p* < 0.01, and ^***^ *p* < 0.001). (**D**) 1205LuP cells were treated with NTP –/+ N-acetyl-cysteine (NAC) for 30 minutes. NTP+NAC is highly significant (^**^ *p* = 0.0036) and NTP+TPZ+NAC is statistically significant (^*^ *p* = 0.0124). Comparisons of single to dual NTP+TPZ treatment were also compared (^*^ *p* < 0.05, ^**^ *p* < 0.01, and ^***^ *p* < 0.001).

Apoptosis was demonstrated with TUNEL assay as induced by NTP, TPZ and highest in NTP+TPZ (Figure 4A). The quantification of apoptosis is shown in Figure 4B with a significantly higher extent of apoptosis in 1205LuC cells with high gap junctions as compared to the vector control (1205LuP) for the conditions of NTP and NTP+TPZ. In addition to apoptosis, oxidative stress levels were measured using the established marker, heme oxygenase-1 (HMOX-1) [30]. The results presented in Figure 4C show that HMOX-1 may play a role because the combination of NTP+TPZ had an additive effect on the induction of HMOX-1, significantly increasing the expression as compared to either agent alone. We also demonstrated that the effect on cell viability was ROS-induced for NTP and NTP+TPZ treatment by showing inhibition with N-acetyl cysteine (NAC) (Figure 4D).

**Figure 5:**
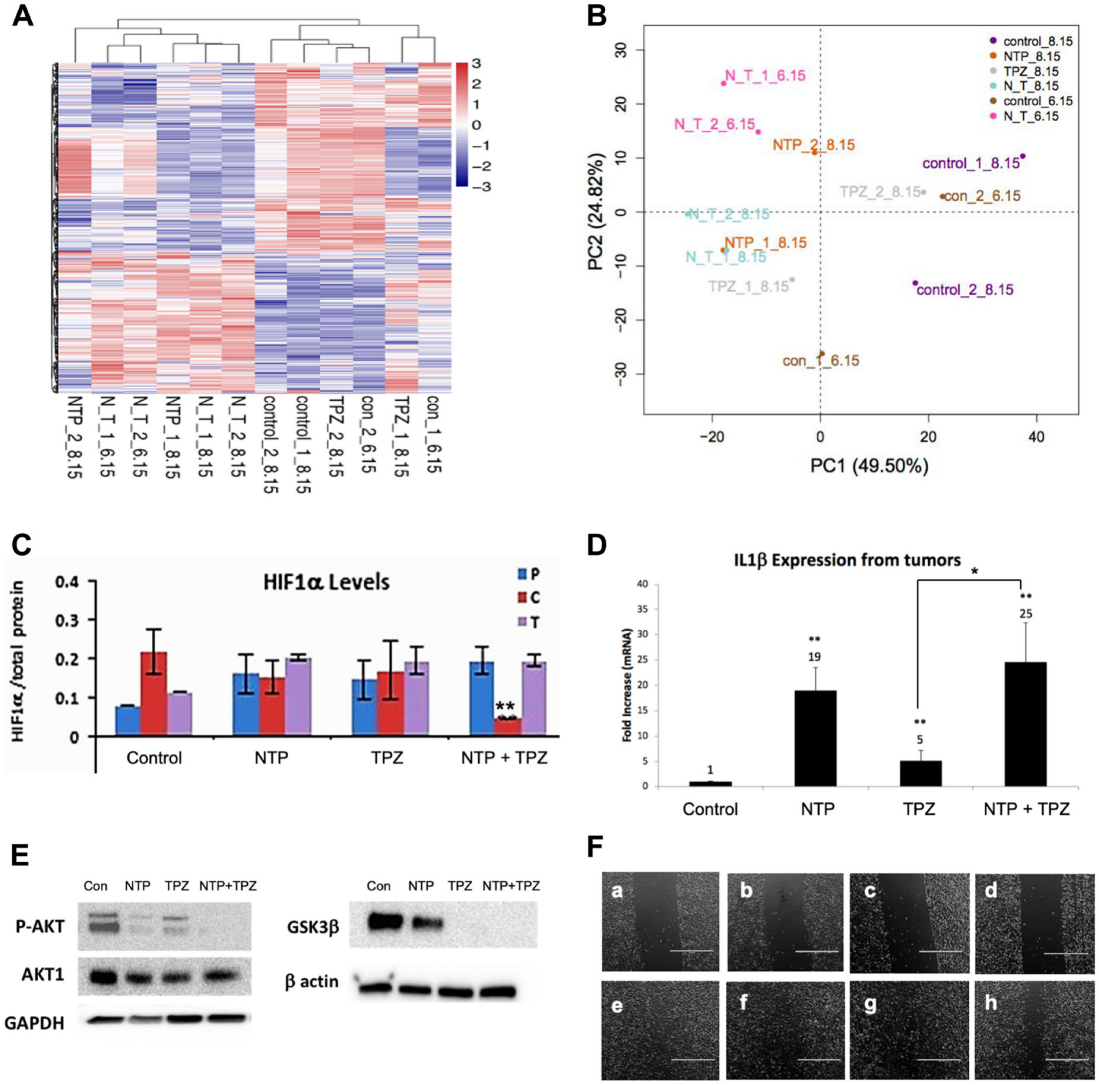
Gene expression changes following NTP+TPZ treatment. (**A**) Clustering Analysis for control, NTP, TPZ, NTP+TPZ treated tumors. (**B**) PCA analysis for control, NTP, TPZ, NTP+TPZ treated tumors. (**C**) HIF-1α levels decrease with NTP+TPZ only in 1205Lu C cells (red) (*p* = 0.0069). (**D**) IL1β expression levels from 1205Lu C tumor lysates treated with NTP, TPZ, or NTP+TPZ. Statistical variances using the unpaired Student’s *t* test are compared to controls unless otherwise indicated (^*^ *p* < 0.05, ^**^ *p* < 0.01, and ^***^ *p* < 0.001). (**E**) Western blot for P-AKT, GSK3β in 1205Lu cells treated with NTP, TPZ, or NTP+TPZ. (**F**) Scratch wounding assay (scale bar = 1000 μm) for Panel a – 1205LuT untreated (time 0); Panel b – 1205LuT TPZ-treated (time 0); Panel c – 1205LuP TPZ-treated (time 0); Panel d – 1205LuC TPZ-treated (time 0); Panel e – 1205LuT untreated (time day 2), percent wound filled = 89.5%; Panel f – 1205LuT TPZ-treated (time day 2), percent wound filled = 82.4%; Panel g – 1205LuP TPZ-treated (time day 2), percent wound filled 84.0%; Panel h – 1205LuC TPZ-treated (time day 2), percent wound filled 62.4%.

Analysis of the microarray results shows a change in multiple genes as demonstrated by the Clustering Analysis of up and down-regulated genes (Figure 5A) and the Principal Component Analysis (PCA) (Figure 5B). Hypoxic conditions are associated with induction of HIF-1α and overexpression of HIF-1α is implicated in initiating angiogenesis, thereby, promoting tumor growth, metastasis and regulation of cellular metabolism to overcome hypoxia [30]. This study demonstrates a decrease in HIF1α protein levels only in melanoma cells treated with NTP+TPZ and expressing high gap junctions (Figure 5C, NTP+TPZ “C”).

There is likely an immunological component to NTP+TPZ therapy since many immune response genes are upregulated (Table 1). The most upregulated gene by microarray was IL1β (25-fold with NTP+TPZ, Figure 5D). Since IL1β is a secreted protein and the tumor samples were rinsed before analysis, the western blot showed a modest protein upregulation of approximately 1.2-fold and did not approximate the levels of the mRNA expression change (Supplementary Figure 4).

**Table 1:** Classification of genes demonstrated to be upregulated or downregulated by NTP+TPZ in the categories of apoptosis, oxidative stress, migration, and immune response

GENE	Log2 FOLD CHANGE	DESCRIPTION	PATHWAY			
			Apoptosis	Oxidative Stress	Migration	Immune Response
ABLIM3	1.10	Actin binding LIM protein family, member 3			X	
AKR1C3	−1.37	Aldo-keto reductase family 1, member C3			X	
ANGPTL7	−1.98	Angiopoietin-like 7		X		
BIRC3	−1.06	Baculoviral IAP repeat-containing 3, transcript variant 1		X		
CAMTA2	−1.29	Calmodulin binding transcription activator 2			X	
CEACAM1	1.10	Carcinoembryonic antigen-related cell adhesion molecule 1 (biliary glycoprotein), transcript variant 2			X	
CTLA4	−1.50	Cytotoxic T-lymphocyte-associated protein 4, transcript variant 1				X
CXCL1	2.85	Chemokine (C-X-C motif) ligand 1 (melanoma growth stimulating activity, alpha)				X
CXCR7	−1.98	Chemokine (C-X-C motif) receptor 7				X
FGF9	1.32	Fibroblast growth factor 9 (glia-activating factor)			X	
FLNC	−1.18	Filamin C, gamma (actin binding protein 280)			X	
GADD45A	−1.10	Growth arrest and DNA-damage-inducible, alpha			X	
GJB2	1.38	Gap junction protein, beta 2, 26kDa	X	X		
IL1B	7.08	Interleukin 1, beta				X
IL6	1.97	Interleukin 6		X		X
IL11	−1.40	Interleukin 11				X
IL12A	−1.57	Interleukin 12A (natural killer cell stimulatory factor 1, cytotoxic lymphocyte maturation factor 1, p35)				X
ITGB8	1.02	Integrin, beta 8			X	
KRT17	1.36	Keratin 17			X	
	2.78	PREDICTED: Similar to Keratin, type I cytoskeletal 14 (Cytokeratin-14) (CK-14) (Keratin-14) (K14) (LOC729252)			X	
MFAP4	1.17	Microfibrillar-associated protein 4			X	
MMP10	1.33	Matrix metallopeptidase 10 (stromelysin 2)			X	
PTGS2	−1.04	Prostaglandin-endoperoxide synthase 2 (prostaglandin G/H synthase and cyclooxygenase)	X			
RRM2B	1.21	Ribonucleotide reductase M2 B (TP53 inducible)		X		
SOD2	1.44	Superoxide dismutase 2, mitochondrial, nuclear gene encoding mitochondrial protein, transcript variant 1	X	X		
SOD3	−1.07	Superoxide dismutase 3, extracellular		X		
TNFRSF1B	1.09	Tumor necrosis factor receptor superfamily, member 1B			X	
ZC3H12A	1.58	Zinc finger CCCH-type containing 12A	X	X		

Effects related to cell migration were also examined via immunoblotting for phospho-AKT and GSK3β, both of which show a decrease in protein expression with NTP alone, TPZ alone, or NTP+TPZ (Figure 5E). Furthermore, with scratch wounding migration assays, the rate of closure of the streaked wound was slowest for the 1205LuC cells (Figure 5F). Table 1 highlights genes with significant up or down-regulation in pathways related to apoptosis, oxidative stress, migration, and the immune response. The results show overall upregulation of genes related to apoptosis, oxidative stress, and the immune response, with down-regulated genes related to enhanced migration and upregulated genes related to cell stability. Further information on these genes are included (Supplementary Table 1).

### Positive feedback via gap junction communication

**Figure 6:**
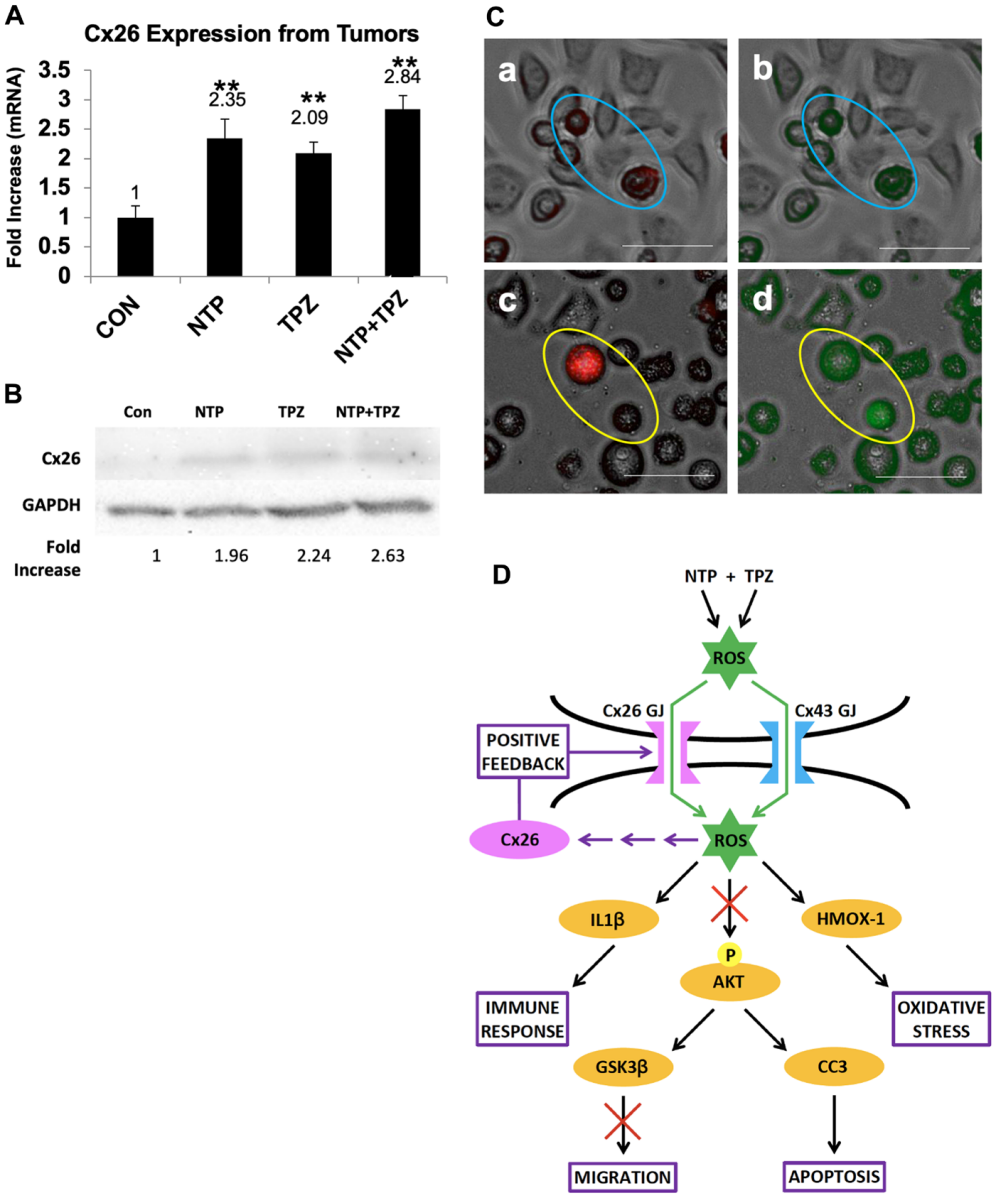
Demonstration of gap junction positive feedback mechanism. (**A**) Analysis of Cx26 mRNA expression from tumors treated with NTP, TPZ, or NTP+TPZ. Statistical variances using the unpaired Student’s *t* test are compared to controls (^*^ *p* < 0.05, ^**^ *p* < 0.01, and ^***^ *p* < 0.001). No significance was determined between single treatments with NTP or TPZ and NTP+TPZ. (**B**) Western blot for HeLa cells treated with NTP, TPZ, or NTP+TPZ as normalized to GAPDH. (**C**) Dye preloading assay in HeLa cells (scale bar = 100 μm) without gap junctions (a, red b, green) in blue ovals; and cells treated with NTP+TPZ (c, red d, green) demonstrating transfer of dye between gap junctions following NTP+TPZ treatment in HeLa cells (yellow ovals). (**D**) Model for the positive feedback mechanism whereby gap junctions are both necessary for the complete NTP+TPZ response and are induced by the therapy.

Connexin 26 was the only GJ gene to be significantly increased in the tumor homogenate microarray analysis (greater than 2-fold) upon treatment with NTP, TPZ, or NTP+TPZ (Figure 6A). Therefore, we specifically wanted to examine the role of Cx26 further. We used wild type (wt) HeLa cells that were previously determined to not express detectable connexin levels [31] as well as a Cx26 specifically expressing variant [32]. By western blot analysis, we demonstrate an increase in Cx26 protein levels in wt HeLa cells treated with NTP, TPZ, or NTP+TPZ (Figure 6B). To test the functionality of the gap junctions, we used the dye preloading assay [33]. This assay utilizes a red dye that is impermeable to gap junctions (DiI) and a green dye (calcein) that is permeable to gap junctions. NTP+TPZ-treated HeLa cells were loaded with both dyes in untreated HeLa cells (Figure 6C, Panel a, b) and NTP+TPZ-treated HeLa cells (Figure 6C, Panel c, d). Only in the treated cells, there was dye transfer from the red cell circled in Panel c to the unlabeled cell circled in Panel c as shown by the green dye transfer in Panel d. This dye transfer occurred prior to the cells rounding up and ultimately dying by the NTP+TPZ treatment. Thus, the combined data from the microarray, western blot, and the preloading assays is indicative of NTP+TPZ-induced increase in Cx26 gap junctions.

A proposed model for the positive feedback mechanism is shown in Figure 6D. ROS molecules and/or their derivatives are suggested to pass through gap junctions between cells to induce cytotoxicity in cancer cells. The molecular permeants induce multiple effects in the cells including increased apoptosis, oxidative stress, increased immune response regulators, and inhibition of migration. Our proposed mechanism for a positive feedback loop is based on the principle that treatment with NTP, TPZ, and NTP+TPZ causes the production of more Cx26 gap junctions. ROS generated from NTP and derivatives of ROS can pass through gap junctions composed of either Cx26 or Cx43 [34]. In addition, TPZ becomes a free radical in hypoxia [35]. TPZ meets the size and charge requirements to pass through gap junctions via the bystander effect [36]. A feedback loop is shown involving ROS, Cx26, and Cx26 gap junctions. The activation of Cx26 is likely to be an indirect effect of ROS, likely through a transcription factor. To this end, Figure 6D includes multiple arrows meant to indicate any number of potential paths that ROS may use to affect the expression of Cx26. The gap junctions amplify the spreading of the ROS signaling effects while inducing increased gap junctions to promote a more potent response.

## DISCUSSION

In this study we demonstrate that NTP causes apoptosis and oxidative stress *in vivo*. We show that TPZ independently causes cell death in melanoma cells under hypoxic conditions. Collectively, NTP+TPZ has an enhanced effect on cell death than either agent alone. Our previous studies have shown that NTP is highly selective for targeting melanoma cells as compared to normal keratinocytes [16]. In addition, it has been established that TPZ undergoes a structural change under low oxygen conditions which induces its cytotoxic activity [19]. Thus, both NTP and TPZ are independently and collectively suited to specifically targeting tumor cells in a hypoxic environment as compared to surrounding stromal cells. This demonstrates a clear advantage of our novel combination therapy as compared to standard chemotherapy which is often associated with high levels of toxicity [22]. In addition to the melanoma cells used in these experiments, we have demonstrated similar results in other melanoma cells (IC_50_ = 26 μM for WM793 premetastatic melanoma cells) as well as other tumor types (Supplementary Figure 1). The likely reason for the high selectivity of the cancer cells is that it has been demonstrated that cancer cells have increased levels of superoxide anion [37, 38]. The principle of our therapeutic treatment is that if tumors are inundated with free radicals and express higher intrinsic levels of free radicals than normal cells, they should reach the threshold for cell death in a highly specific manner.

The most compelling aspect of this research is the potential to affect cancer cells distal to the site of treatment. This is accomplished through the bystander effect whereby upregulation of gap junctions promotes the intercellular transfer of molecules that causes cell death [24]. Gap junctions enable the transfer of molecules up to 1 kDa between cells, including ROS. Non-thermal plasma concentrates multiple charged and uncharged species emanating from ionized gases. The non-thermal plasma utilized in these studies emits 45 species, many of which are ROS [12]. Furthermore, TPZ itself acts as a ROS molecule only when it undergoes a conformational change induced by hypoxia [19]. Due to the short half-life of ROS molecules, it is more likely that more stable derivatives of ROS are the actual permeants through the gap junctions. Treatment with both non-thermal plasma and drug therapy enables ROS molecules (and/or downstream molecules that induce apoptosis) to pass through gap junctions, increasing the surface area of treatment, thus promoting the apoptotic cell death of the tumor.

By microarray analysis of human metastatic melanoma tumors treated with NTP+TPZ, we have identified multiple genes and pathways regulated by this therapy. We have shown a decrease in HIF-1α protein levels only under conditions of NTP+TPZ and high gap junctions. HIF-1α overexpression is associated with tumor aggression for several types including glioblastoma, oligodendroglioma, melanoma, breast, cervical, colon, ovarian, endometrial, lung, prostate, bladder, pancreatic, and oropharyngeal cancer. Therapies targeting HIF-1α are being rapidly investigated [39]. This is the first report of gap junctions playing a role in regulating HIF-1α.

In addition to the upregulation of apoptosis and apoptosis-inducing pathways [40], oxidative stress genes are also regulated such as HMOX-1. HMOX-1 is a microsomal enzyme that is both induced in response to cellular stress and acts to suppress oxidative stress by reduction of ROS generation [41]. HMOX-1 was shown to inhibit migration and progression to mesenchymal transition (EMT) [42]. This may explain why the results demonstrate a decrease in gene expression associated with migration and a reduced ability to migrate in a streak wounding assay (Figure 5E, 5F). In addition to oxidative stress induction, we observed an increase in expression of interleukins and cytokines. IL1β was shown to be the most upregulated gene of the microarray analysis (Figure 5B). In A549 lung adenocarcinoma cells, nonthermal plasma was shown to enhance the anti-tumor activity of macrophages by releasing proinflammatory cytokines including IL1β to cause tumoricidal activity [43]. Other cytokines and chemokines are differentially regulated include CTLA4, CXCL1, CXCR7, IL6, AND IL12A (see Table 1) This immune response coupled with the specificity of the NTP+TPZ therapy suggests a potential alternative to standard immunotherapy [5, 6] with less side effects on the patient.

This study demonstrates that the role of gap junctions is necessary for the large extent of cell death in melanoma with NTP+TPZ. However, tumor development is often associated with low levels of gap junctions [44]. In some tumor types such as breast, lung, prostate, and others, gap junctions upregulate in the cells as they become metastatic [45–47]. However, the timing of the upregulation of the gap junctions and the treatment protocol may not coincide. Several inhibitors of gap junctions are commonly utilized experimentally or clinically such as carbenoxolone, quinidine, oleamide, heptanol, octanol, glycyrrhetinic acid, and retinoic acid [48]. Activators of gap junctions, such as tedisamil, have undergone phase III studies for cardiac arrhythmias [49]. However, a high selectivity for gap junction upregulation in tumor tissue must be maintained to prevent cardiac arrhythmias and seizures [48]. We have developed a combination therapy with both high specificity for tumor cells and the ability to upregulate Cx26 gap junctions [16].

Although tumor regression occurred in mouse xenografts for both high (1205LuC) and low gap junction-expressing (1205LuP) models, the results were more accentuated in the high gap junction-expressing model. Interestingly, our studies show that tumors from both of these groups regress for at least 6 days after the treatment was discontinued. We show, for the first time, that NTP+TPZ reduces tumor volume and promotes apoptosis which is enhanced by gap junctions. The therapy itself induces the expression and functionality of Cx26 gap junctions. Since gap junctions are both enhancers of the therapeutic effect and become induced by the therapy, we propose that the combination therapy activates a positive feedback mechanism. Future studies will be directed at pursuing the long-term effects of the combination NTP+TPZ therapy *in vivo* to potentially develop a novel treatment for melanoma as well as other tumor types.

## MATERIALS AND METHODS

### Cell culture

The 1205Lu cells (Wistar Institute, Philadelphia, PA, USA) transfected with PBMN-IRES-GFP (1205LuP) with wild type Cx43 (1205LuC) or dominant negative Cx43 (1205LuT) have been previously described [27]. HeLa and HeLa26 cells were described [50]. HCT116, HCT8, WB793B (ATCC). All cells were cultured in DMEM (Invitrogen, Grand Island, NY, USA) media supplemented with 10% FBS, 1% pen/strep. For all experimental conditions with TPZ, cells were kept in a hypoxic chamber (Biospherix Model^#^E702) within a cell incubator, regulating the oxygen level at 0.1% O_2_.

### NTP and TPZ treatments

NTP treatments were performed at the following conditions: 147-200 kHz with a flow rate of 3 L/min and a distance of 1 cm from the source. This study used TPZ (Sigma, St. Louis, MO, USA) with a structure as reported [18]. Cells were treated with TPZ at a serial dilution to determine the IC_50_. The drug was initially dissolved in 0.0001% DMSO and the dose for treatment was determined accordingly, diluted in cell culture media. The experiments were conducted as follows: day 1: plate cells in 12 well plates at 2 × 10^5^ cells/well; day 2: change media +/– TPZ (hypoxic chamber); day 3: remove media, treat +/– plasma, add DMEM without TPZ, return to hypoxic chamber; day 4 assess results (microscopy, viability assay with presto blue (Invitrogen, cat#A13262) and plate reader (BioTek Synergy HT, Winooski, VA, USA). Cells were also treated with NTP +/– N-acetyl-cysteine (NAC) (Sigma) at 100 μM NAC for 30 minutes.

### Quantitative real-time PCR

Experiments were performed with Real-Time PCR for HMOX1 as normalized to β-actin with numerical values only, so there are no gel bands available. Total cellular RNA was isolated using the RNeasy Mini Kit (QIAGEN, Hilden, Germany). Quantitative PCR was prepared using cDNA Reverse Transcription kit (Invitrogen). Quantitative RT-PCR was performed on 2900HT Fast Real-Time PCR System (Applied Biosystems, Foster City, CA, USA) using TaqMan Universal Master Mix II (Applied Biosystems). PCR data were analyzed using Sequence Detection Software 2.4 (Applied Biosystems). All reactions were performed in triplicate, and the experiments were repeated at least twice.

### Protein expression analysis

#### Western blot

Membranes were developed with alkaline phosphatase-conjugated secondary antibodies, and signals were visualized using either the Alpha Innotech FluorChem HD2 Imaging System (San Leandro, CA, USA) or the FluoroChem M system (Cell Biosciences, Preston, Australia). The following antibodies were purchased from Santa Cruz Biotechnology (Dallas, TX, USA): Cx26 (sc-130729), Il1β (sc-52012), P-AKT (sc-7985R), AKT (sc-5298), GSK3β (sc-81462), and β-actin (sc-47778). GAPDH (#14C10) was from Cell Signaling Technology (Danvers, MA, USA). Primary antibodies were used at a 1:1000 dilution with Santa Cruz HRP-conjugated anti- mouse and anti-rabbit secondary antibodies at 1:3000. We tested both the change in protein expression by western blot and the change in the extent of coupling by the dye preloading assay which is a functional assay for dye transfer between cells. We utilized parental HeLa cells that do not express gap junctions and HeLa cells that are stably transfected with Cx26 [31]. We have characterized HeLa26 cells according to growth and migration properties [32, 50].

#### Protein assay

HIF1α protein levels were determined with an ELISA kit ab117996 (Abcam, Cambridge, UK). These values were divided by the total cell number to obtain a value of HIF1α protein levels per total cell number.

### TUNEL assay

TUNEL assay was performed according to established protocols on sectioned formalin fixed tumor tissue (Millipore Sigma, St. Louis, MO, USA) [51]. Briefly, tissues were permeabilized using ethanol to allow for penetration of the TUNEL reaction reagents into the cell nucleus. Following fixation and washing, incorporation of biotinylated-UTP onto the three prime ends of fragmented DNA was carried out in the reaction containing TdT enzyme.

### Microarray

Gene expression changes were analyzed by a microarray of 30,000 cancer signaling-related genes at Roswell Park Comprehensive Cancer Center (RPCCC, Buffalo, NY, USA). All microarray data was generated using Illumina’s Human HT-12 v4 Expression Bead Chip (San Diego, CA, USA). The data was processed using the neqc method implemented in R (version 3.2.2) package limma. It performs (a) background correction, (b) quantile normalization and (c) expression level quantification based on log2 transformation. Expression levels of multiple probes corresponding to the same gene were averaged to represent the expression level of the gene.

Differential expression analysis was performed in the following four comparisons: control versus NTP, control versus N_T and control versus TPZ for experiment 1; control versus N_T for experiment 2. Multiple log2FC cut-offs (|log2FC|>2, |log2FC|>1) were adopted to select significantly up- and down-regulated genes. The differentially expressed genes selected by cutoff |log2FC| >1 were used for overlapped up- and down-regulated gene finding across four comparisons clustering analysis, Principal Component Analysis (PCA) and pathway analysis. Hierarchical clustering analysis was performed using differentially expressed genes. The analysis was carried out using the function pheatmap in R (version 3.2.2) package Pheatmap1 (version 1.0.7). Configuration: clustering_distance_rows = “euclidean”, clustering_distance_cols = “euclidean”, clustering_method = “complete”. PCA was carried out using function pca in R (version 3.2.2) package FactoMineR2 (version 1.31.4) with differentially expressed gene. Configuration: scale. unit = TRUE, ncp = 5, ind. sup = NULL, quanti. sup = NULL, quali. sup = NULL, row. w = NULL, col. w = NULL, graph = TRUE, axes = c (1,2).

### Mouse tumor studies

All mouse studies were approved by IACUC protocol #1111 at RPCCC. All mice were purchased from Jackson Laboratories (Bar Harbor, ME, USA). Human metastatic melanoma cells were implanted in the flanks subcutaneously of severe combined immunodeficient (SCID) mice as xenograft experiments. Cells from 1205LuP and 1205LuC were injected subcutaneously into the flanks of the mice at 1 × 10^6^ cells per flank with two injections per mouse. Once the tumors reached 150 cubed millimeters (mm^3^), treatments with the combination therapy were delivered every two to three days for a total of five treatments. NTP treatment was given as described above with two 30-second treatments given at each application to the mouse skin surface. TPZ was injected at a concentration of 20 μM with a volume of 100 microliter (μL) per injection. In experiments with NTP alone (Figure 1A), NTP treatment was applied to the skin surface every two days up to day 10 (Figure 1A). In another experiment, (Supplementary Figure 3), we compared saline injected tumors to untreated tumors, showing no statistical significance. In a third experimental series (Figure 3C and 3D), the tumors were either untreated, or treated with NTP, TPZ, or NTP+TPZ. In all three experiments noted above, the tumors were measured every two days with calipers until day 10. Then, from day 10 to day 16, the mice were untreated. At day 16, the tumors were measured, then excised and either fixed in 10% formalin for histological analysis or frozen at –80°C for protein and RNA analysis. In these experiments, five mice were used per condition with two tumors per mouse for a total of 10 tumors analyzed per condition. The experiment was repeated for three independent experiments. Statistical analysis was performed using the unpaired student *t*-test with alpha set at 0.05.

### Scratch wounding assay

Each well was plated with 3 × 10^5^ cells per 35 mm dish for 1205LuP, 1205LuC, and 1205LuT cells. After 24 hours (day = 0), a pipette tip from a 1000 μL Pipetman was used to scratch a line through the middle of the plate. Every 24 hours, the cell images were taken and compared (data shown = day 2). The percent of the wound filled by day two was analyzed using the area under the curve setting on the ImageJ program.

### Dye preloading assay

The assay was performed according to the referenced protocol [31]. Briefly, HeLa cells were used because they do not express any detectable gap junctions [48]. The TPZ and NTP were treated as described above. Cells were incubated with two dyes, calcein, a green gap junction permeable dye (Invitrogen, Carlsbad, CA, USA), and DiI, a red gap junction impermeable dye (Invitrogen). The cells were mixed with unlabeled cells at a ratio of 1:50. Following time to allow cells to adhere (five hours), images were taken, identifying cells with red dye and then observing the extent of gap junction communication through the surrounding cells labelled in green.

## SUPPLEMENTARY MATERIALS



## Abbreviations

BRAF: v-Raf murine sarcoma viral oncogene homolog B
MEK: mitogen-activated protein kinase kinase
CTLA-4: cytotoxic T-lymphocyte associated protein 4
PD-1: phosphodiesterase 1
NTP: non-thermal plasma
TPZ: tirapazamine
GJIC: gap junction intercellular communication
DBD: dielectrode barrier diffusion
ROS: reactive oxygen species
DMEM: Dulbecco’s modified Eagle medium
FBS: fetal bovine serum
TUNEL: terminal deoxynucleotidyl transferase nick end labeling
CC3: cleaved caspase 3
NRF2: nuclear factor erythroid 2-related factor-2
TXNRD2: thioredoxin reductase 2
SCID: severe combined immunodeficiency
HMOX-1: heme oxygenase 1
HIF-1: hypoxia inducible factor 1
EMT: epithelial mesenchymal transition
NAC: N-acetyl-cysteine

## References

[1] American Cancer Society. Statistical Analysis. Retrieved Jan 2020 URL: http://www.cancer.org.

[2] Scholtens A , Foppen MG , Blank CU , van Thienen JV , van Tinteren H , Haanen JB . Vemurafenib for BRAF V600 mutated advanced melanoma: results of treatment beyond progression. Eur J Cancer. 2015; 51:642–652. 10.1016/j.ejca.2015.01.009. 25690538

[3] Ascierto PA , McArthur GA , Dréno B , Atkinson V , Liszkay G , Di Giacomo AM , Mandalà M , Demidov L , Stroyakovskiy D , Thomas L , de la Cruz-Merino L , Dutriaux C , Garbe C , et al. Cobimetinib combined with vemurafenib in advanced BRAF(V600)-mutant melanoma (coBRIM): updated efficacy results from a randomised, double-blind, phase 3 trial. Lancet Oncol. 2016; 17:1248–2360. 10.1016/S1470-2045(16)30122-X. 27480103

[4] Ribas A , Gonzalez R , Pavlick A , Hamid O , Gajewski TF , Daud A , Flaherty L , Logan T , Chmielowski B , Lewis K , Kee D , Boasberg P , Yin M , et al. Combination of vemurafenib and cobimetinib in patients with advanced BRAF(V600)-mutated melanoma: a phase 1b study. Lancet Oncol. 2014; 15:954–965. 10.1016/S1470-2045(14)70301-8. 25037139

[5] Wei SC , Levine JH , Cogdill AP , Zhao Y , Anang NA , Andrews MC , Sharma P , Wang J , Wargo JA , Pe’er D , Allison JP . Distinct cellular mechanisms underlie anti-CTLA-4 and anti-PD-1 checkpoint blockade. Cell. 2017; 170:1120–1133.e17. 10.1016/j.cell.2017.07.024. 28803728 PMC5591072

[6] Ulloa-Montoya F , Louahed J , Dizier B , Gruselle O , Spiessens B , Lehmann FF , Suciu S , Kruit WH , Eggermont AM , Vansteenkiste J , Brichard VG . Predictive gene signature in MAGE-A3 antigen-specific Cancer immunotherapy. J Clin Oncol. 2013; 31:2388–2395. 10.1200/JCO.2012.44.3762. 23715562

[7] Zucker S . U.S. Patent No. 9,586,056. Washington, DC: U.S. Patent and Trademark Office. 2017.

[8] Zirnheld JL , Zucker SN , DiSanto TM , Berezney R , Etemadi K . Nonthermal plasma needle: development and targeting of melanoma cells. IEEE Trans Plasma Sci. 2010; 38:948–952. 10.1109/TPS.2010.2041470.

[9] Kajiyama H , Utsumi F , Nakamura K , Tanaka H , Toyokuni S , Hori M , Kikkawa F . Future perspective of strategic non-thermal plasma therapy for cancer treatment. J Clin Biochem Nutr. 2017; 60:33–38. 10.3164/jcbn.16-65. 28163380 PMC5281532

[10] Fridman G , Shereshevsky A , Jost MM , Brooks AD , Fridman A , Gutsol A , Vasilets V , Friedman G . Floating electrode dielectric barrier discharge plasma in air promoting apoptotic behavior in melanoma skin cancer cell lines. Plasma Chem Plasma Process. 2007; 27:163–176. 10.1007/s11090-007-9048-4.

[11] Zirnheld JL , DiSanto TM , Burke KM , Zucker SN , Etemadi K . Development of an atmospheric pressure non-thermal plasma needle for melanoma cell research. [conference paper] In: Proceedings of the IEEE Pulsed Power Conference; Jun 28-Jul 2, 2009; Washington, DC. Washington, DC: IEEE Conference Record 2009; 17:Abstract nr O21-27.

[12] Sakiyama Y , Graves DB . Neutral gas flow and ring-shaped emission profile in non-thermal RF-excited plasma needle discharge at atmospheric pressure. Plasma Sources Sci Technol. 2009; 18:25022–25033. 10.1088/0963-0252/18/2/025022.

[13] Niemi K , O’Connell D , de Oliveira N , Joyeux D , Nahon L , Booth JP , Gans T . Absolute atomic oxygen and nitrogen densities in radio-frequency driven atmospheric pressure cold plasmas: synchrotron vacuum ultra-violet high-resolution Fourier-transform absorption measurements. Appl Phys Lett. 2013; 103:034102 10.1063/1.4813817.

[14] Yalcin S , Marinkovic D , Mungamuri SK , Zhang X , Tong W , Sellers R , Ghaffari S . ROS-mediated amplification of AKT/mTOR signalling pathway leads to myeloproliferative syndrome in Foxo3(-/-) mice. EMBO J. 2010; 29:4118–31. 10.1038/emboj.2010.292. 21113129 PMC3018793

[15] Ciccarese F , Ciminale V . Escaping death: mitochondrial redox homeostasis in cancer cells. Front Oncol. 2017; 7:A117. 10.3389/fonc.2017.00117. 28649560 PMC5465272

[16] Zucker SN , Zirnheld J , Bagati A , DiSanto TM , Des Soye B , Wawrzyniak JA , Etemadi K , Nikiforov M , Berezney R . Preferential induction of apoptotic cell death in melanoma cells as compared with normal keratinocytes using a non-thermal plasma torch. Cancer Biol Ther. 2012; 13:1299–1306. 10.4161/cbt.21787. 22895073 PMC3493438

[17] Phillips RM . Targeting the hypoxic fraction of tumors using hypoxia-activated prodrugs. Cancer Chemother Pharmacol. 2016; 77:441–457. 10.1007/s00280-015-2920-7. 26811177 PMC4767869

[18] Shinde SS , Hay MP , Patterson AV , Denny WA , Anderson RF . Spin trapping of radicals other than the *OH radical upon reduction of the anticancer agent tirapazamine by cytochrome 450 reductase. J Am Chem Soc. 2009; 131:14220–14221. 10.1021/ja906860a. 19772319

[19] Brown JM , Wilson WR . Exploiting tumor hypoxia in cancer treatment. Nat Rev Cancer. 2004; 4:437–447. 10.1038/nrc1367. 15170446

[20] U.S. National Library of Medicine. https://clinicaltrials.gov. Retrieved Jan 2020 URL: https://clinicaltrials.gov/ct2/results?term=tirapazamine&Search=Apply&age_v=&gndr=&type=&rslt=&phase=1&phase=2.

[21] McKeown SR , Cowen RL , Williams KJ . Bioreductive drugs: from concept to clinic. Clin Oncol (R Coll Radiol). 2007; 19:427–442. 10.1016/j.clon.2007.03.006. 17482438

[22] Lexicomp Online, Lexi-Drugs Online, Hudson, Ohio: Wolters Kluwer Clinical Drug Information, Inc.; retrieved Jan 2020.

[23] Sosinsky GE , Nicholson BJ . Structural organization of gap junction channels. Biochim Biophys Acta. 2005; 1711:99–125. 10.1016/j.bbamem.2005.04.001. 15925321

[24] Zhang D , Zhou T , He F , Rong Y , Lee SH , Wu S , Zuo L . Reactive oxygen species formation and bystander effects in gradient irradiation on human breast cancer cells. Oncotarget. 2016; 7:41622–41636. 10.18632/oncotarget.9517. 27223435 PMC5173083

[25] Arora S , Heyza JR , Chalfin EC , Ruch RJ , Patrick SM . Gap junction intercellular communication positively regulates cisplatin toxicity by inducing DNA damage through bystander signaling. Cancers (Basel). 2018; 10:368. 10.3390/cancers10100368. 30279363 PMC6210410

[26] Beahm DL , Oshima A , Gaietta GM , Hand GM , Smock AE , Zucker SN , Toloue MM , Chandrasekhar A , Nicholson BJ , Sosinsky GE . Mutation of a conserved threonine in the third transmembrane helix of alpha- and beta-connexins creates a dominant-negative closed gap junction channel. J Biol Chem. 2006; 281:7994–8009. 10.1074/jbc.M506533200. 16407179

[27] Zucker SN , Bancroft TA , Place DE , Des Soye B , Bagati A , Berezney R . A dominant negative Cx43 mutant differentially affects tumorigenic and invasive properties in human metastatic melanoma cells. J Cell Physiol. 2013; 228:853–859. 10.1002/jcp.24235. 23042412

[28] Thompson K , Burke K , Zirnheld J , Zucker SN . Comparison of circuits for generating nonthermal plasma to treat melanoma. IEEE Trans Dielectr Electr Insul. 2017; 24:2241–2247. 10.1109/TDEI.2017.006288.

[29] Olbryt M , Habryka A , Tyszkiewicz T , Rusin A , Cichoń T , Jarząb M , Krawczyk Z . Melanoma-associated genes, MXI1, FN1, and NME1, are hypoxia responsive in murine and human melanoma cells. Melanoma Res. 2011; 21:417–425. 10.1097/CMR.0b013e328348db2f. 21912348

[30] Zimna A , Kurpisz M . Hypoxia-inducible factor-1 in physiological and pathophysiological angiogenesis: applications and therapies. Biomed Res Int. 2015; 2015:549412. 10.1155/2015/549412. 26146622 PMC4471260

[31] Elfgang C , Eckert R , Lichtenberg-Fraté H , Butterweck A , Traub O , Klein RA , Hülser DF , Willecke K . Specific permeability and selective formation of gap junction channels in connexin-transfected HeLa cells. J Cell Biol. 1995; 129:805–817. 10.1083/jcb.129.3.805. 7537274 PMC2120441

[32] Chandrasekhar A , Kalmykov EA , Polusani SR , Mathis SA , Zucker SN , Nicolson BJ . Intercellular redistribution of cAMP underlies selective suppression of cancer cell growth by connexin 26. PLoS One. 2013; 8:e82335. 10.1371/journal.pone.0082335. 24312655 PMC3849486

[33] Goldberg GS , Bechberger JF , Naus CC . A pre-loading method of evaluating gap junctional communication by fluorescent dye transfer. Biotechniques. 1995; 18:490–497. 7779401

[34] Huang Y , Mao Z , Zhang Z , Obata F , Yang X , Zhang X , Huang Y , Mitsui T , Fan J , Takeda M , Yao J . Connexin43 contributes to inflammasome activation and lipopolysaccharide-initiated acute renal injury via modulation of intracellular oxidative status. Antioxid Redox Signal. 2019; 31:1194–1212. 10.1089/ars.2018.7636. 31319679

[35] Yang B , Keshelava N , Anderson CP , Reynolds CP . Antagonism of buthionine sulfoximine cytotoxicity for human neuroblastoma cell lines by hypoxia is reversed by the bioreductive agent tirapazamine. Cancer Res. 2003; 63:1520–1526. 12670899

[36] Jella KK , Moriarty R , McClean B , Byrne HJ , Lyng FM . Reactive oxygen species and nitric oxide signaling in bystander cells. PLoS One. 2018; 13:e0195371. 10.1371/journal.pone.0195371. 29621312 PMC5886541

[37] Valko M , Rhodes CJ , Moncol J , Izakovic M , Mazur M . Free radicals, metals and antioxidants in oxidative stress-induced cancer. Chem Biol Interact. 2006; 160:1–40. 10.1016/j.cbi.2005.12.009. 16430879

[38] Locatelli C , Leal PC , Yunes RA , Nunes RJ , Crecynski-Pasa TB . Gallic acid ester derivatives induce apoptosis and cell adhesion inhibition in melanoma cells: the relationship between free radical generation, glutathione depletion and cell death. Chem Biol Interact. 2009; 181:175–184. 10.1016/j.cbi.2009.06.019. 19577552

[39] Garvalov BK , Acker T . Implications of oxygen homeostasis for tumor biology and treatment. Adv Exp Med Biol. 2016; 903:169–185. 10.1007/978-1-4899-7678-9_12. 27343096

[40] Hu Q , Peng J , Liu W , He X , Cui L , Chen X , Huang Y , Mitsui T , Fan J , Takeda M , Yao J . Elevated cleaved caspase-3 is associated with shortened overall survival in several cancer types. Int J Clin Exp Pathol. 2014; 7:5057–5070. 25197379 PMC4152069

[41] Jozkowicz A , Was H , Dulak J . Heme oxygenase-1 in tumors: is it a false friend. Antioxid Redox Signal. 2007; 9:2099–2117. 10.1089/ars.2007.1659. 17822372 PMC2096718

[42] Gueron G , Giudice J , Valacco P , Paez A , Elguero B , Toscani M , Jaworski F , Leskow FC , Cotignola J , Marti M , Binaghi M , Navone N , Vazquez E . Heme-oxygenase-1 implications in cell morphology and the adhesive behavior of prostate cancer cells. Oncotarget. 2014; 5:4087–4102. 10.18632/oncotarget.1826. 24961479 PMC4147308

[43] Lin A , Truong B , Patel S , Kaushik N , Choi EH , Fridman G , Fridman A , Miller V . Nanosecond-pulsed DBD plasma-generated reactive oxygen species trigger immunogenic cell death in A549 lung carcinoma cells through intracellular oxidative stress. Int J Mol Sci. 2017; 18:966. 10.3390/ijms18050966. 28467380 PMC5454879

[44] Naus CC , Laird DW . Implications and challenges of connexin connections to cancer. Nat Rev Cancer. 2010; 10:435–441. 10.1038/nrc2841. 20495577

[45] Kanczuga-Koda L , Sulkowski S , Lenczewski A , Koda M , Wincewicz A , Baltaziak M , Sulkowska M . Increased expression of connexins 26 and 43 in lymph node metastases of breast cancer. J Clin Pathw. 2006; 59:429–433. 10.1136/jcp.2005.029272. 16567471 PMC1860373

[46] Ezumi K , Yamamoto H , Murata K , Higashiyama M , Damdinsuren B , Nakamura Y , Kyo N , Okami J , Ngan CY , Takemasa I , Ikeda M , Sekimoto M , Matsuura N , et al. Aberrant expression of connexin 26 is associated with lung metastasis of colorectal cancer. Clin Cancer Res. 2008; 14:677–684. 10.1158/1078-0432.CCR-07-1184. 18245526

[47] Tate AW , Lung T , Radhakrishnan A , Lim SD , Lin X , Edlund M . Changes in gap junctional connexin isoforms during prostate cancer progression. Prostate. 2006; 66:19–31. 10.1002/pros.20317. 16114058

[48] Manjarrez-Marmolejo J , Franco-Pérez J . Gap junction blockers: an overview of their effects on induced seizures in animal models. Curr Neuropharmacol. 2016; 14:759–771. 10.2174/1570159X14666160603115942. 27262601 PMC5050393

[49] De Mello WC , Thormahlen D . Effect of tedisamil on cell communication, impulse propagation, and excitability of the failing heart. Eur J Pharmacol. 1999; 372:241–246. 10.1016/S0014-2999(99)00199-5. 10395018

[50] Polusani SR , Kalmykov EA , Chandrasekhar A , Zucker SN , Nicholson BJ . Cell coupling mediated by connexin 26 selectively contributes to reduced adhesivity and increased migration. J Cell Sci. 2016; 129:4399–4410. 10.1242/jcs.185017. 27777264 PMC5201008

[51] Loo DT . *In situ* detection of apoptosis by the TUNEL assay: an overview of techniques . Methods Mol Biol. 2011; 682:3–13. 10.1007/978-1-60327-409-8_1. 21057916

