# Supplementary material for: Novel combination therapy for melanoma induces apoptosis via a gap junction positive feedback mechanism
Source: Oncotarget. 2020 Sep 15;11(37):3443–58. doi: 10.18632/oncotarget.27732 (PMC7500108; doi:10.18632/oncotarget.27732)
Supplement: Supplementary file 1 [file oncotarget-11-3443-s001.pdf]

Novel combination therapy for melanoma induces apoptosis via a gap junction positive feedback mechanism

SUPPLEMENTARY MATERIALS

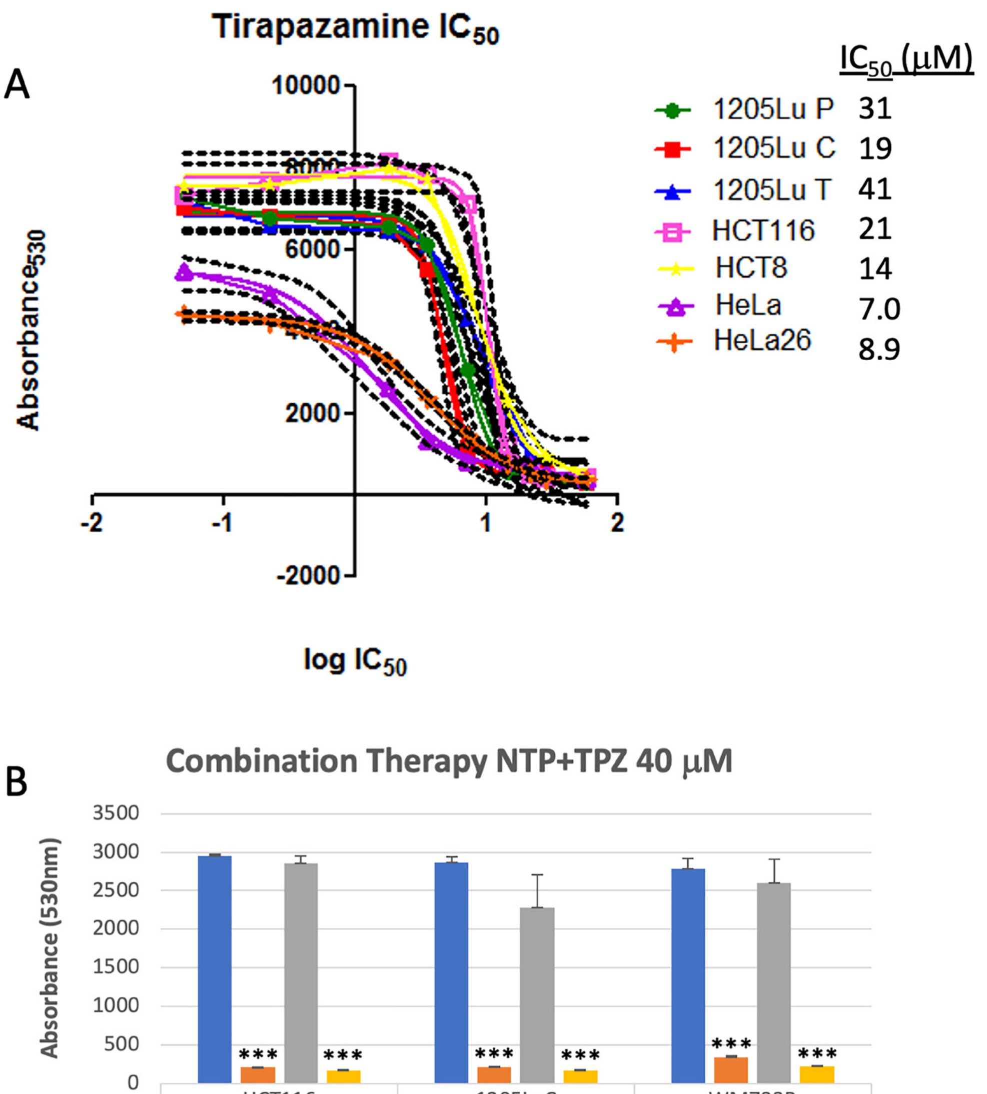

	HCT116	1205LuC	WM793B
control	2948	2866	2786
TPZ	206	213	340
NTP	2860	2285	2604
NTP+TPZ	165	163	223

Supplementary Figure 1: IC_{50} for several cell lines with TPZ. (**A**) HCT116 and HCT8 are colon cancer cell lines. HeLa and HeLa26 are cervical carcinoma cell lines. HeLa26 is a stable transfectant of HeLa. (**B**) Assay for cell viability with NTP, TPZ, NTP+TPZ (*** indicates $p < 0.001$). There is no statistical significance between TPZ and NTP+TPZ.

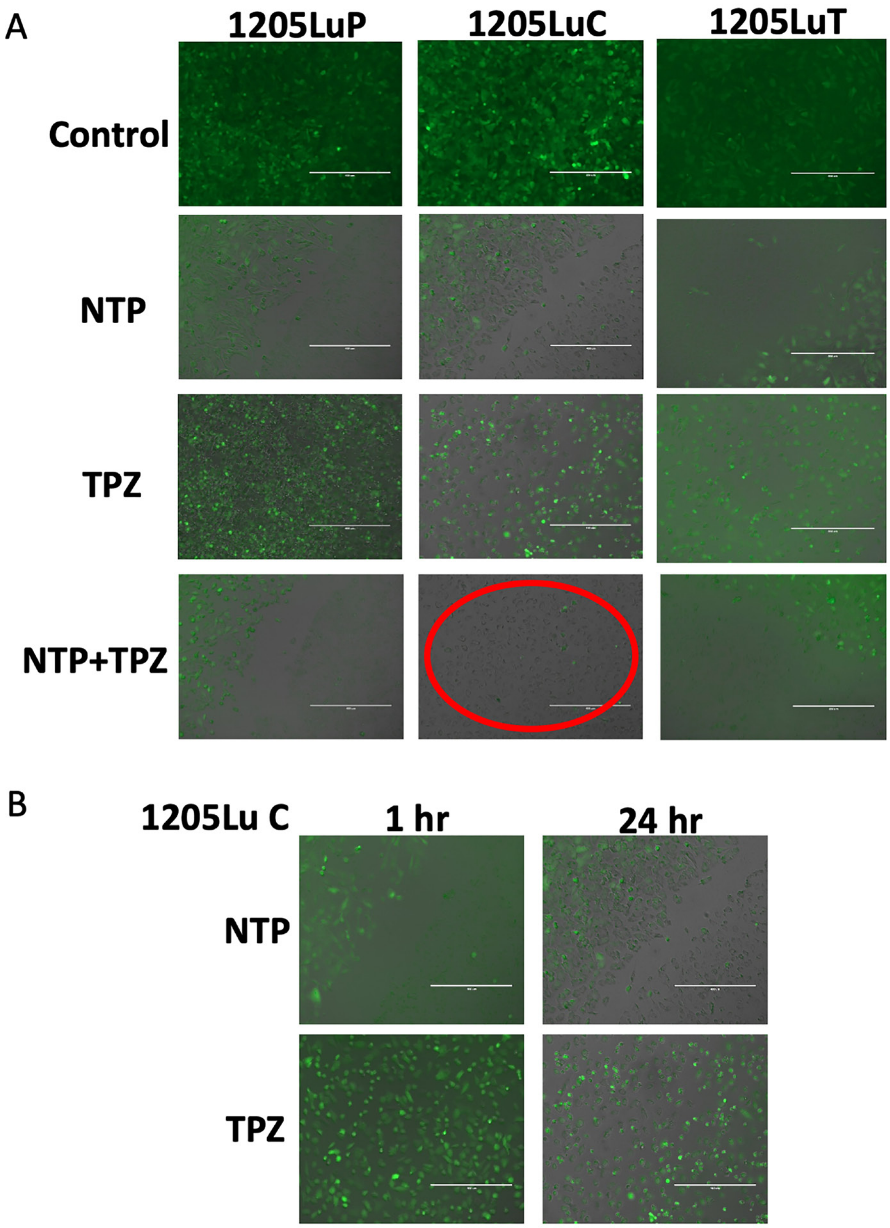

Supplementary Figure 2: Gap junction expression increases cell death in 1205LuC cells. (**A**) The GFP which has been stably transfected into all three cell lines loses its expression upon cell death; this is most apparent in 1205LuC cells treated with NTP + TPZ (red circle). These images were taken following 24 hours in hypoxia post-treatment. (**B**) Comparison of extent of green cells (viability) at 1 hour and 24 hours post-treatment in 1205LuC cells (scale bar = 400 µm).

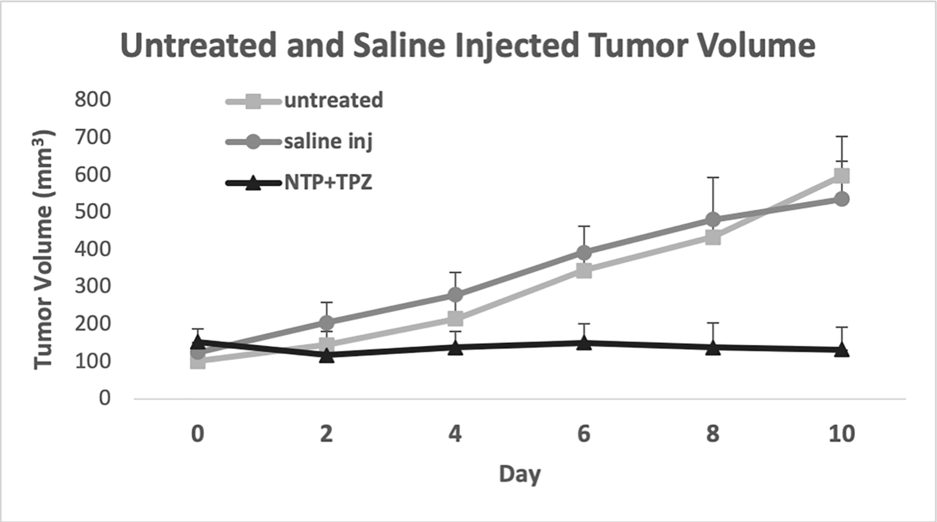

Supplementary Figure 3: Comparison of growth for tumor xenografts either untreated or saline injected. Tumor volume for 1205LuC xenografts from SCID mice was measured in every 2 days to compare the effects of untreated and saline injected tumors. There is no statistical significance between untreated and saline injected, yet both conditions are highly significant as compared to NTP+TPZ by day 10 ($p < 0.0001$).

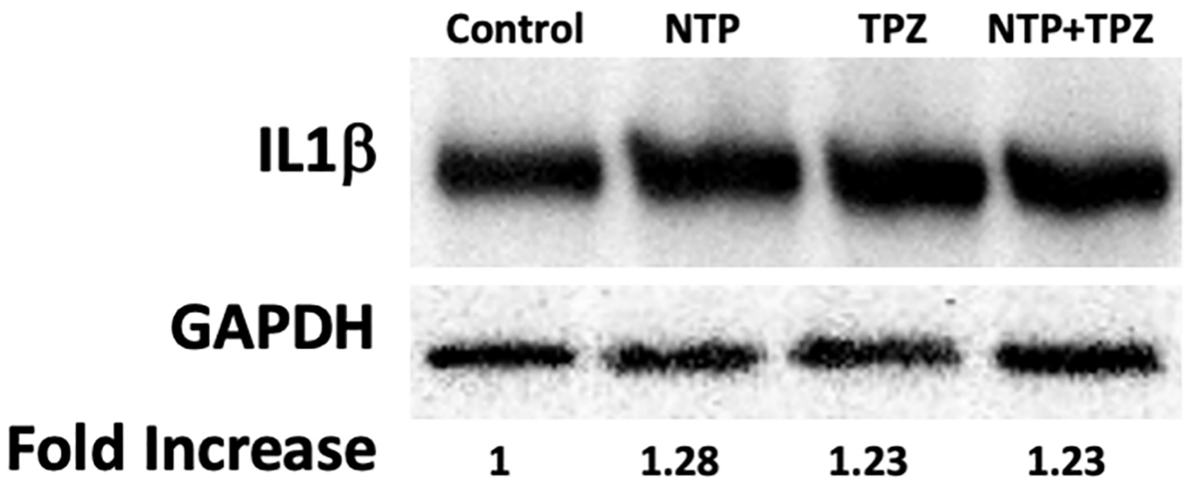

Supplementary Figure 4: Western blot for IL1β normalized to GAPDH.

Supplementary Table 1: Description of genes modified by treatment with NTP+TPZ according to microarray analysis

Gene	Functional Pathway
ABLIM3	ABLIM3 promotes protein-protein interaction. High expression found to increase invasiveness of hepatoblastoma [1].
AKR1C3	AKR1C3 contributes to breast cancer development through its 17B-HSD and PGF2a synthase activities. AKR1C3 also plays a pivotal role in all pathways of androgen biosynthesis in prostate cancer by catalyzing the pathway to DHT [2, 3].
ANGPTL7	ANGPTL7 is over-expressed in different human cancers and is marginally expressed under standard growth condition while it is specifically up-regulated by hypoxia. It also exerts a pro-angiogenetic effect on human differentiated endothelial cells [4].
BIRC3	BIRC3 induces tumor proliferation and metastasis *in vitro* and *in vivo* [5].
CAMTA2	Calmodulin-binding transcription activator protein found to impact cardiac growth. CAMTA2 is found to be under-expressed in certain pancreatic cancers [6, 7].
CEACAM1	CEACAM1 is highly expressed in malignant melanomas. The interaction of CEACAM1 with other CEA families are involved in influencing survival and apoptosis, migration, invasion, and modulation of immune responses [8, 9].
CTLA4	CTLA4 functions as inhibitory receptors on T-cells to inhibit tumor apoptosis and cytotoxic T cell action [10].
CXCL1	High levels of CXCL1 in tumor tissues and serum are correlated with worse prognosis in several tumor types due to the preferential recruitment of pro-tumorigenic immune cells via the CXCR1/2 axis [11].
CXCR7	CXCR7 expression is controlled by the particular microenvironment of tumors. The transcriptional and translational levels are increased in hypoxic conditions in glioma cell lines and promote migration. The expression of CXCR7 restricts the ability for paracrine regulation [12, 13].
FGF9	FGF9 acts as a possible mediator secreted by cancer-associated fibroblasts that promotes the anti-apoptosis and invasive capabilities of cancer cells through activation of the ERK and Akt signaling pathways [14].
FLNC	FilaminC, an actin binding and cross-linking filamin protein gene, has been found to increase invasiveness and migration of many cancers including prostate cancer, glioblastomas, and esophageal cancer [15–17].
GADD45A	GADD45A is a stress sensor in pulmonary embolism, and it is a predicted target of miR-376c in trophoblasts. DLX6 Antisense 1 are seen to be overexpressed in rats in their neurons within the subventricular zone of the developing forebrain. Migration and invasion capabilities are able to increase in cells without GADD45A [18].
GJB2	Gene responsible for gap junction production. In cells with expression of GJB2, cell cycle arrest, downregulation of BCL2 expression, and apoptosis were observed. Mutations of this gene causes apoptosis and oxidative stress within the inner ear, causing deafness [19–21].
1L1B	1L1B is shown to affect innate and adaptive immune response by increasing immunity markers and encoding Th2 marks making inflammation a greater response and having increased SNPs [22].
IL6	IL6 has a role in immune response which includes variability among individuals and tumors in the major histocompatibility complex molecules. The response has shown to increase annexin II derived peptides that are bound to MHC class II molecules. Expression of MHC molecules in cancer are induced as a result of cytokine secretion by tumor infiltrating cells. The inflammatory cytokines contribute to increased expression of MHC molecules and to annexin I and II translocation to the cell surface, resulting in a response by the humoral immune response. IL6 has a direct role in oxidative stress by mediating the transcriptional effects; a signaling cascade of ROS (i.e., H2O2), IL-6, and phosphorylated p38 is proposed for regulating effects on matrix degradation [23, 24].
IL12A	IL12A has proven causality for any SNP variant, as it has shown to have a clear function in immune response. Specific pathways involve inducing chemokine and cytokines, and B and T Cell activation [25].
ITGB8	ITGB8 restores cancer cell migration and invasion by increasing cell proliferation and colony formation, thus ITGB8 functions as an oncogenic gene in cancer cell growth and metastasis [26].
KRT17	Selective knock down of KRT17 by RNA interference could significantly supress cell proliferation, inhibit the cell migration and reduce tumor growth *in vivo* [27].

SUPPLEMENTARY REFERENCES

1. Krupp M, Weinmann A, Galle PR, Teufel A. "Actin binding LIM protein 3 (abLIM3)". International Journal of Molecular Medicine. 2006; 17:129–33. https://doi.org/10.3892/ijmm.17.1.129. [PubMed]
2. Jansson AK, Gunnarsson C, Cohen M, Sivik T, Stål O. 17β-Hydroxysteroid Dehydrogenase 14 Affects Estradiol Levels in Breast Cancer Cells and Is a Prognostic Marker in Estrogen Receptor–Positive Breast Cancer. Cancer research. 2006; 66:11471–11477. https://doi.org/10.1158/0008-5472.CAN-06-1448. [PubMed]
3. Penning TM. AKR1C3 (Type 5 17β-hydroxysteroid dehydrogenase/prostaglandin F synthase): Roles in malignancy and endocrine disorders. Mol Cell Endocrinol. 2019; 489:82–91. https://doi.org/10.1016/j.mce.2018.07.002. [PubMed]
4. Parri M, Pietrovito L, Grandi A, Campagnoli S, De Camilli E, Bianchini F, Marchiò S, Bussolino F, Jin B, Sarmientos P, Grandi G, Viale G, Pileri P, et al. Angiopoietin-like 7, a novel pro-angiogenetic factor over-expressed in cancer. Angiogenesis. 2014; 17:881–896. https://doi.org/10.1007/s10456-014-9435-4. [PubMed]
5. Fu PY, Hu B, Ma XL, Yang ZF, Yu MC, Sun HX, Huang A, Zhang X, Wang J, Hu ZQ, Zhou CH, Tang WG, Ning R, et al. New insight into BIRC3: A novel prognostic indicator and a potential therapeutic target for liver cancer. J Cell Biochem. 2019; 120:6035–6045. https://doi.org/10.1002/jcb.27890. [PubMed]
6. Zhang CL, Mckinsey TA, Chang S, Antos CL, Hill JA, Olson EN. Class II Histone Deacetylases Act as Signal-Responsive Repressors of Cardiac Hypertrophy. Cell. 2002; 110:479–488. https://doi.org/10.1016/s0092-8674(02)00861-9. [PubMed]
7. Liang JW, Shi ZZ, Shen TY, Che X, Wang Z, Shi SS, Xu X, Cai Y, Zhao P, Wang CF, Zhou ZX, Wang MR. Identification of genomic alterations in pancreatic cancer using array-based comparative genomic hybridization. PLoS One. 2014; 9:e114616. https://doi.org/10.1371/journal.pone.0114616. [PubMed]
8. Ebrahimnejad A, Streichert T, Nollau P, Horst AK, Wagener C, Bamberger AM, Brümmer J. CEACAM1 enhances invasion and migration of melanocytic and melanoma cells. Am J Pathol. 2004; 165:1781–1787. https://doi.org/10.1016/S0002-9440(10)63433-5. [PubMed]
9. Beauchemin N, Arabzadeh A. Carcinoembryonic antigen-related cell adhesion molecules (CEACAMs) in cancer progression and metastasis. Cancer Metastasis Rev. 2013; 32:643–671. https://doi.org/10.1007/s10555-013-9444-6. [PubMed]
10. Karlsson AK, Saleh SN. Checkpoint inhibitors for malignant melanoma: a systematic review and meta-analysis. Clin Cosmet Investig Dermatol. 2017; 10:325–339. https://doi.org/10.2147/CCID.S120877. [PubMed]
11. Susek KH, Karvouni M, Alici E, Lundqvist A. The role of CXC chemokine receptors 1–4 on immune cells in the tumor microenvironment. Front Immunol. 2018; 9:2159. https://doi.org/10.3389/fimmu.2018.02159. [PubMed]
12. Esencay M, Sarfraz Y, Zagzag D. CXCR7 is induced by hypoxia and mediates glioma cell migration towards SDF-1α. BMC cancer. 2013; 13:347. https://doi.org/10.1186/1471-2407-13-347. [PubMed]
13. Wang C, Chen W, Shen J. CXCR7 targeting and its major disease relevance. Front Pharmacol. 2018; 9:641. https://doi.org/10.3389/fphar.2018.00641. [PubMed]
14. Sun C, Fukui H, Hara K, Zhang X, Kitayama Y, Eda H, Tomita T, Oshima T, Kikuchi S, Watari J, Sasako M, Miwa H. FGF9 from cancer-associated fibroblasts is a possible mediator of invasion and anti-apoptosis of gastric cancer cells. BMC Cancer. 2015; 15:333. https://doi.org/10.1186/s12885-015-1353-3. [PubMed]
15. Tanabe K, Shinsato Y, Furukawa T, Kita Y, Hatanaka K, Minami K, Kawahara K, Yamamoto M, Baba K, Mori S, Uchikado Y, Maemura K, Tanimoto A, Natsugoe S. Filamin C promotes lymphatic invasion and lymphatic metastasis and increases cell motility by regulating Rho GTPase in esophageal squamous cell carcinoma. Oncotarget. 2017; 8:6353–6363. https://doi.org/10.18632/oncotarget.14087. [PubMed]
16. Ai J, Jin T, Yang L, Wei Q, Yang Y, Li H, Zhu Y. Vinculin and filamin-C are two potential prognostic biomarkers and therapeutic targets for prostate cancer cell migration. Oncotarget. 2017; 8:82430–82436. https://doi.org/10.18632/oncotarget.19397. [PubMed]
17. Kamil M, Shinsato Y, Higa N, Hirano T, Idogawa M, Takajo T, Minami K, Shimokawa M, Yamamoto M, Kawahara K, Yonezawa H, Hirano H, Furukawa T, et al. High filamin-C expression predicts enhanced invasiveness and poor outcome in glioblastoma multiforme. Br J Cancer. 2019; 120:819–826. https://doi.org/10.1038/s41416-019-0413-x. [PubMed]
18. Tan Y, Xiao D, Xu Y, Wang C. Long non-coding RNA DLX6-AS1 is upregulated in preeclampsia and modulates migration and invasion of trophoblasts through the miR-376c/GADD45A axis. Exp Cell Res. 2018; 370:718–724. https://doi.org/10.1016/j.yexcr.2018.07.039. [PubMed]
19. Tanaka M, Grossman HB. Connexin 26 gene therapy of human bladder cancer: induction of growth suppression, apoptosis, and synergy with Cisplatin. Hum Gene Ther. 2001; 12:2225–2236. https://doi.org/10.1089/10430340152710568. [PubMed]
20. Bilal S. Structure modeling and mutational analysis of gap junction beta 2 (GJB2). Afr J Biotechnol. 2012; 11:6965–6973. https://doi.org/10.5897/ajb11.3540.
21. Fetoni AR, Zorzi V, Paciello F, Ziraldo G, Peres C, Raspa M, Scavizzi F, Salvatore AM, Crispino G, Tognola G, Gentile G, Spampinato AG, Cuccaro D, et al. Cx26 partial loss causes accelerated presbycusis by redox imbalance and

dysregulation of Nfr2 pathway. Redox Biol. 2018; 19:301–317. https://doi.org/10.1016/j.redox.2018.08.002. [PubMed]

22. Wang MH, Helzlsouer KJ, Smith MW, Hoffman-Bolton JA, Clipp SL, Grinberg V, De Marzo AM, Isaacs WB, Drake CG, Shugart YY, Platz EA. Association of IL10 and other immune response-and obesity-related genes with prostate cancer in CLUE II. Prostate. 2009; 69:874–885. https://doi.org/10.1002/pros.20933. [PubMed]

23. Brichory FM, Misek DE, Yim AM, Krause MC, Giordano TJ, Beer DG, Hanash SM. An immune response manifested by the common occurrence of annexins I and II autoantibodies and high circulating levels of IL-6 in lung cancer. Proc Natl Acad Sci U S A. 2001; 98:9824–9829. https://doi.org/10.1073/pnas.171320598. [PubMed]

24. Nieto N. Oxidative-stress and IL-6 mediate the fibrogenic effects of rodent Kupffer cells on stellate cells. Hepatology. 2006; 44:1487–1501. https://doi.org/10.1002/hep.21427. [PubMed]

25. Hunt KA, Zhernakova A, Turner G, Heap GA, Franke L, Bruinenberg M, Romanos J, Dinesen LC, Ryan AW, Panesar D, Gwilliam R, Takeuchi F, McLaren WM, et al. Newly identified genetic risk variants for celiac disease related to the immune response. Nat Genet. 2008; 40:395–402. https://doi.org/10.1038/ng.102. [PubMed]

26. Huang L, Cai JL, Huang PZ, Kang L, Huang MJ, Wang L, Wang JP. miR19b-3p promotes the growth and metastasis of colorectal cancer via directly targeting ITGB8. Am J Cancer Res. 2017; 7:1996–2008. [PubMed]

27. Chivu-Economescu M, Dragu DL, Necula LG, Matei L, Enciu AM, Bleotu C, Diaconu CC. Knockdown of KRT17 by siRNA induces antitumoral effects on gastric cancer cells. Gastric Cancer. 2017; 20:948–959. https://doi.org/10.1007/s10120-017-0712-y. [PubMed]
